# Dual Metabolic Inhibition by Berberine and Glutor Triggers AMPK/JNK-Dependent DNA Damage in Cancer Cells

**DOI:** 10.7150/ijms.132977

**Published:** 2026-05-29

**Authors:** Meina Shi, Chengming Wei, Dayuan Zheng, Dongfan Yang, Tong Chu, Shaokui Liang, Lu Yang, Kuanyun Zhang, Yanchao Yang, Wenzhe Ma

**Affiliations:** 1 State Key Laboratory of Mechanism and Quality of Chinese Medicine & Faculty of Chinese Medicine, Macau University of Science and Technology, Macau SAR, 999078, China.; 2 Zhuhai MUST Science and Technology Research Institute, Macau University of Science and Technology, Hengqin Guangdong-Macao In-Depth Cooperation Zone, Guangdong, 519099, China.; 3 Department of Surgery, School of Clinical Medicine, LKS Faculty of Medicine, The University of Hong Kong, Queen Mary Hospital, Hong Kong SAR, 999077, China.

**Keywords:** Berberine, Glutor, JNK, ATP, DNA damage

## Abstract

Metabolic reprogramming is a key adaptive feature of malignant tumors and often leads to the failure of therapeutic strategies targeting a single metabolic pathway. In this study, we investigated the antitumor activity of combining berberine (BBR), a mitochondrial complex I inhibitor, with Glutor, a pan-glucose transporter inhibitor that blocks glucose uptake across multiple GLUT isoforms. The combination produced strong synergistic antiproliferative effects in HeLa, HepG2, and HCT116 cells, with Bliss synergy scores of 45.967, 34.219, and 26.972, respectively. Co-administration simultaneously suppressed glycolysis and oxidative phosphorylation (OXPHOS), resulting in severe ATP depletion and persistent activation of the AMPK signaling pathway, thereby inducing pronounced metabolic stress. Further mechanistic investigations revealed that such sustained metabolic stress effectively activated the JNK signaling pathway, which in turn exacerbated DNA damage, impaired homologous recombination repair, and ultimately triggered caspase-dependent apoptosis. Pharmacological inhibition or genetic silencing of JNK attenuated DNA damage and partially restored HR repair-related proteins, supporting JNK as a key functional mediator of the downstream stress response. In *ex vivo* tumor explant models, the combination also showed cooperative antitumor activity. These findings indicate that simultaneous restriction of glucose uptake/glycolysis and mitochondrial oxidative phosphorylation, termed “dual metabolic inhibition” cooperatively impairs tumor cell survival. The AMPK/JNK axis appears to be a critical mechanistic link underlying this antitumor effect.

## 1. Introduction

Metabolic reprogramming is a hallmark of malignant tumors, and the Warburg effect represents a central feature of this process. The Warburg effect refers to the preferential reliance of cancer cells on glycolysis for energy production, even under aerobic conditions 1. Inhibition of glucose transport into tumor cells can effectively block this energy-acquisition pathway, thereby suppressing tumor cell growth. A wealth of studies has demonstrated that the glucose transporter (GLUT) family plays a central role in regulating transmembrane glucose transport, and functional inhibition of these transporters can effectively constrain the Warburg effect 2. Consequently, GLUT family inhibitors have garnered significant attention as a potential anticancer strategy. Glutor is a potent pan-GLUT inhibitor with reported preferential activity against GLUT1 and GLUT3. At nanomolar concentrations, it effectively impedes glucose uptake and glycolysis in tumor cells and selectively inhibits the proliferation of various cancer cell lines 3. Beyond directly inhibiting glucose transport, Glutor exerts antitumor effects through multiple mechanisms, such as downregulating key metabolic enzymes including hexokinase-2 (HK-2) and lactate dehydrogenase A (LDHA), and inducing mitochondrial dysfunction and apoptosis by modulating the HIF-1α/p53/Bcl-2 signaling axis 4. These findings underscore the significant antitumor potential of Glutor, warranting further investigation and clinical exploration. However, tumor cell metabolism exhibits considerable plasticity. Solely inhibiting glycolysis can trigger compensatory metabolic reprogramming 5, 6, rendering strategies targeting only glucose uptake insufficient for sustained tumor suppression. Previous studies have confirmed that Glutor combined with the glutaminase inhibitor CB-839 produces a marked synergistic antitumor effect 7. Therefore, combining glucose uptake inhibitors with other targeted agents holds promise for achieving more effective antitumor outcomes.

Berberine (BBR), an isoquinoline alkaloid derived from *Coptis chinensis*, has attracted widespread interest in oncology therapeutics by virtue of its multi-target pharmacological profile. Beyond regulating multiple signaling pathways, including AMPK/mTOR, PI3K/AKT, TLR4/NF-κB and MAPK 8, 9, a central aspect of its antitumor activity involves reprogramming cellular energy metabolism, particularly by targeting mitochondrial OXPHOS 10. Specifically, BBR inhibits mitochondrial respiratory chain complex I (NADH dehydrogenase), thereby suppressing OXPHOS, reducing cellular oxygen consumption rate (OCR) and ATP production, and elevating the AMP/ATP ratio. This metabolic shift activates the AMPK signaling pathway, initiates metabolic reprogramming, and ultimately results in cell death. This mechanism has been validated in multiple preclinical models, including non-alcoholic fatty liver disease associated hepatocellular carcinoma, glioma, colorectal cancer, gastrointestinal cancer and pancreatic cancer 11-15. Beyond its direct inhibitory effects, BBR can also indirectly suppress the activity of mitochondrial complex I by facilitating the ubiquitination and proteasomal degradation of SIRT3 16. These properties further support its potential for combination therapy with other anticancer agents. For instance, co-administration of BBR combined with doxorubicin reverses chemoresistance in breast cancer cells by activating the AMPK signaling pathway and repressing HIF-1α 17, whereas its combination with emodin potently inhibits glycolysis and enhances apoptosis in breast cancer models 18. Previous studies have also shown that BBR combined with 2-deoxy-D-glucose (2-DG) simultaneously blocks glycolysis and OXPHOS, causing severe ATP depletion and cell death in lung cancer cells 19. However, the present strategy is conceptually distinct from the previously reported BBR + 2-DG combination. Specifically, Glutor inhibits glucose transport at the level of nutrient entry by targeting multiple GLUT isoforms, whereas 2-DG acts after glucose uptake and primarily interferes with intracellular glucose metabolism. This upstream intervention provides a mechanistically distinct approach to dual metabolic restriction and may impose a stronger constraint on tumor metabolic compensation. In addition, because 2-DG is known to affect processes beyond glycolysis inhibition, including N-linked glycosylation and ER stress, the BBR-Glutor combination may allow clearer interpretation of downstream stress responses.

Importantly, BBR was not selected merely as an AMPK-activating agent. Rather, it was chosen as a mitochondrial OXPHOS-disrupting compound whose bioenergetic effects complement the upstream blockade of glucose entry mediated by Glutor. While Glutor limits glucose availability by inhibiting multiple GLUT isoforms at the level of nutrient entry, BBR suppresses mitochondrial respiration through inhibition of complex I 14, 20. We therefore reasoned that this combination could simultaneously restrict two major and partially compensatory bioenergetic pathways, namely glucose uptake/glycolysis and mitochondrial oxidative phosphorylation 21. In this study, we refer to this strategy as “dual metabolic inhibition”, which is expected to limit reciprocal metabolic compensation more effectively than either intervention alone. Based on this rationale, we hypothesized that co-treatment with Glutor and BBR would exacerbate bioenergetic stress, activate downstream stress signaling pathways, and ultimately produce synergistic antitumor effects.

This study provides a systematic investigation into the synergistic antitumor activity of the combination of Glutor and BBR, along with its underlying molecular mechanism. In HeLa, HepG2, and HCT116 cell lines, the combined treatment synergistically inhibited both glycolysis and OXPHOS, thereby inducing energy stress, persistently activating the AMPK/JNK pathway, exacerbating DNA damage, and suppressing HR repair, ultimately driving tumor cell apoptosis. Our work elucidates a synergistic antitumor mechanism mediated by dual metabolic inhibition, and offers a translationally promising approach to surmount tumor metabolic plasticity and clinical therapeutic resistance, which holds the potential to underpin the advancement of more potent combinatorial anticancer therapies.

## 2. Materials and Methods

### 2.1 Cell Lines and Culture Conditions

The human cancer cell lines HeLa (cervical adenocarcinoma), HepG2 (hepatocellular carcinoma), and HCT116 (colorectal carcinoma), as well as the non-malignant/immortalized human cell models AC16 cardiomyocyte-like cells and L02 hepatic cells, were obtained from the American Type Culture Collection (ATCC). HeLa, HepG2, HCT116, and L02 cells were cultured in Dulbecco's modified Eagle's medium (DMEM; Gibco), whereas AC16 cells were cultured in Dulbecco's modified Eagle's medium/F-12 (DMEM/F-12; Gibco). All culture media were supplemented with 10% fetal bovine serum (FBS; Gibco), 100 U/mL penicillin, and 100 μg/mL streptomycin (Sigma-Aldrich). Cells were maintained at 37°C in a humidified incubator with 5% CO₂ and passaged at 80-90% confluency using 0.25% trypsin-EDTA (Gibco).

### 2.2 Chemicals

BBR (CAS: 2086-83-1) and SP600125 (CAS: 129-56-6) were purchased from Dalian Meilun Biotechnology Co., Ltd. (Dalian, China) and dissolved in dimethyl sulfoxide (DMSO; Acros Organics, Morris Plains, NJ, USA) for storage at -40 °C. Puromycin (CAS: 53-79-2), sulforhodamine B (SRB; CAS: 3520-42-1), trichloroacetic acid (TCA; CAS: 76-03-9), tris-base (CAS: 77-86-1), N-acetyl-L-cysteine (NAC; CAS: 616-91-1), and crystal violet (CAS: 548-62-9) were obtained from Sigma-Aldrich. Q-VD-OPh (HY-12305), SB202190 (HY-10295), chloroquine (CQ; HY-17589A), Ferrostatin-1 (Fer-1; HY-100579), Liproxstatin-1 (Lip-1; HY-12726), BSJ-04-122 (HY-152185), and Glutor (HY-158302) were purchased from MedChemExpress (Monmouth Junction, NJ, USA). Unless otherwise indicated, all compounds were dissolved in DMSO, and an equal volume of DMSO was added to control cells as the vehicle control in all experiments.

### 2.3 Antibodies

Antibodies used for western blotting are listed below by supplier. Primary antibodies: cleaved caspase-3 (#9664), cleaved caspase-9 (#9505), cleaved PARP (#5625), JNK (#9252), Bim (#2933), phospho-JNK (Thr183/Tyr185; #9255), phospho-AMPK (Thr172; #2535), phospho-MKK4 (Ser257/Thr261; #9156), and phospho-p38 MAPK (Thr180/Tyr182; #4511) (all rabbit monoclonal except cleaved caspase-9, which is rabbit polyclonal; Cell Signaling Technology); CtIP (sc-271339), Ku70 (sc-17789), and TopBP1 (sc-271043) (all mouse monoclonal; Santa Cruz Biotechnology); γ-H2AX (phospho-S139; 05-636) (mouse monoclonal; Thermo Scientific); RAD51 (HY-P80297) (rabbit monoclonal; MedChemExpress); DNA-PKcs (ET1610-12) (rabbit monoclonal; HUABIO); β-actin (A5441) (mouse monoclonal; Sigma-Aldrich). Secondary antibodies: HRP-conjugated anti-mouse (715-035-150) and anti-rabbit (211-035-109) (Jackson ImmunoResearch).

### 2.4 Cell Proliferation Assay

Cell proliferation was assessed using the sulforhodamine B (SRB) assay. HeLa, HepG2, and HCT116 cells were seeded in 96-well plates at 7,000 cells per well. After overnight adhesion, cells were treated with serial dilutions of the compounds or DMSO vehicle for 24 or 48 h. Cells were then fixed with cold 10% trichloroacetic acid for 1 h, stained with 0.4% SRB for 30 min, washed with 1% acetic acid, and the bound dye was solubilized in 10 mM Tris-base. Absorbance was measured at 515 nm using a microplate reader 22. Cell viability was calculated relative to vehicle controls, and IC50 values were determined by nonlinear regression analysis in GraphPad Prism 8.0 (GraphPad Software, San Diego, CA, USA).

### 2.5 Drug Combination and Synergy Analysis

To assess drug interaction effects, HeLa, HepG2, and HCT116 cells were seeded in 96-well plates (7,000 cells/well) and allowed to adhere overnight. Cells were then treated with serially diluted BBR and Glutor, either individually or in combination, for 24 or 48 h. Drug interactions were evaluated using the Bliss independence model implemented in SynergyFinder. Synergy scores were classified as follows: < -10 (antagonism), -10 to 10 (additivity), and > 10 (synergy). The results are presented as two- and three-dimensional heatmaps, with synergistic and antagonistic dose regions colored red and green, respectively 23.

### 2.6 Colony Formation Assay

Cells were plated in 6-well plates at 2,000 cells per well. After 2 days of culture, cells were treated with BBR (4 μM) alone, Glutor (4 nM) alone, or their combination for 8 days. Colonies were fixed, stained with crystal violet, and imaged using a Gel Doc XR system (Bio-Rad).

### 2.7 Annexin V/7-AAD Apoptosis Assay

Apoptosis was assessed by flow cytometry (BD FACSAria II) with an Annexin V-APC/7-AAD staining kit (Keygen Biotech, KGA1106). Cells were seeded in 6-well plates and allowed to adhere for 24 h before treatment with the indicated compounds. After 24 h of drug exposure, cells were harvested and resuspended in binding buffer containing Annexin V-APC and 7-AAD for 10 min at room temperature in the dark. A total of 10,000 events were acquired per sample within 1 h and analyzed using FlowJo software (v10.4.0) to determine the percentage of cells in early and late apoptotic stages.

### 2.8 Western Blot Analysis

Total proteins were extracted with RIPA buffer containing protease/phosphatase inhibitors. Following BCA quantification, equal amounts of protein were resolved by SDS-PAGE, transferred to PVDF membranes, and probed with primary and HRP-conjugated secondary antibodies. Blots were developed with chemiluminescent substrate and imaged using an Amersham Imager 600 (GE Healthcare). All Western blot experiments were independently repeated at least three times. The blot images shown in the figures were selected as representative of the consistent expression patterns observed across independent experiments.

### 2.9 Intracellular ATP Measurement

Intracellular ATP levels in HeLa and HepG2 cells were determined after 24 h of drug treatment using a luminescence-based viability assay (CellTiter-Lumi^TM^, Beyotime). Following reagent addition and cell lysis, luminescent signals were recorded with a microplate reader (SpectraMax Paradigm, Molecular Devices). ATP levels were normalized to cell number and expressed as relative luminescence units (RLU). Experiments were performed in triplicate with three independent biological replicates.

### 2.10 Comet Assay

DNA damage was assessed using a Comet Assay Kit (Beyotime) according to the manufacturer's instructions. Briefly, cells were harvested and resuspended in 1% low-melting-point agarose, then embedded on slides pre-coated with 1% normal-melting-point agarose. Electrophoresis was performed in neutral electrophoresis buffer. DNA was stained with propidium iodide (PI) and visualized under a fluorescence microscope (Olympus IX71). Images were analyzed using ImageJ software with the Comet Assay Software Project plugin (Beijing Biolaunching Technologies). DNA damage was quantified by the Olive Tail Moment (OTM), calculated as: OTM = (Tail._mean_ - Head._mean_) × (Tail %DNA/100).

### 2.11 Lentiviral Transduction and Stable Cell Line Generation

JNK knockdown was achieved using shRNA lentiviral vectors. The target-specific oligonucleotide sequences were: shJNK#1 sense, 5′-TGAAAGAATGTCCTACCTTCT-3′; antisense, 5′-AGAAGGTAGGACATTCTTTCA-3′ (MiaoLingBio, Wuhan, China). Annealed duplexes were cloned into the pLKO.1 vector via AgeI/EcoRI sites, and sequences were verified by Sanger sequencing. For lentivirus production, HEK293T cells at 70% confluency were transfected with 0.5 µg pLKO.1-shJNK (or NC-shRNA), 75 ng psPAX2 (Addgene #12260), and 25 ng pMD2.G (Addgene #12259) using FuGENE HD (Promega) in Opti-MEM. Viral supernatants were collected at 24 h and 48 h post-transfection, filtered through 0.45 µm PES membranes, and stored at -80 °C. Target cells were transduced in the presence of polybrene for 24 h, then selected with puromycin for 72 h. Knockdown efficiency was validated by Western blotting.

### 2.12 Mitochondrial Membrane Potential Assay

Mitochondrial membrane potential (ΔΨm) was determined utilizing the JC-1 dye (MCE, HY-K0601). Post-treatment for 24 h, cells were incubated with JC-1 in the dark. Red fluorescence (Ex/Em 560/595 nm), resulting from the formation of J-aggregates in polarized mitochondria, was contrasted with green fluorescence (Ex/Em 485/535 nm), derived from cytosolic monomers in depolarized mitochondria. ΔΨm depolarization was quantified as the percentage of JC-1 green-positive cells by flow cytometry.

### 2.13 Glucose Uptake Assay

Cellular glucose levels were determined using a colorimetric assay kit (Beyotime, S0201S). After treatment, cells were lysed and cleared supernatants were analyzed according to the manufacturer's protocol. Absorbance readings were taken at 630 nm, and glucose concentrations were calculated from a standard curve and normalized to total protein. Experiments were performed in triplicate, and statistical analysis was conducted using one-way ANOVA in GraphPad Prism 8.0.

### 2.14 Lactate Production Assay

Extracellular lactate levels were quantified in culture supernatants using a colorimetric L-lactic acid assay kit (Elabscience) following the manufacturer's instructions. Absorbance was measured at 530 nm and lactate concentrations were calculated against a sodium lactate standard curve. Values were normalized to total protein content and expressed as percentage lactate production.

### 2.15 Bioinformatic Analysis

Publicly available transcriptomic and clinical data were analyzed to establish the clinical relevance of targeting GLUTs and to select appropriate cancer models for this study. Using the TIMER 2.0 database (http://timer.comp-genomics.org), we assessed the differential mRNA expression of SLC2A1 (GLUT1) and SLC2A3 (GLUT3) between tumor tissues and matched adjacent normal tissues across multiple cancer types from The Cancer Genome Atlas (TCGA). All databases were accessed with standardized normalization protocols to ensure cross-dataset comparability.

### 2.16 Proteomics Analysis

HCT116 cells were treated with DMSO (control), BBR (8 µM), Glutor (16 nM), or their combination for 24 h. Cells were lysed in 8 M urea, and protein concentration was determined by BCA assay. Proteins (100 µg per sample) were reduced with TCEP, alkylated with IAM, and digested overnight with trypsin (1:50). Peptides were desalted and analyzed on a Vanquish Neo LC system coupled to an Orbitrap Astral mass spectrometer in data-independent acquisition (DIA) mode (m/z 100-1700). Raw data were processed using Spectronaut, requiring ≥ 3 unique peptides per protein and an FDR ≤ 0.01. Bioinformatics analysis was performed on the Majorbio Cloud platform (https://cloud.majorbio.com). Differentially expressed proteins (DEPs) were defined as fold change > 2 or < 0.5 with p < 0.05. Pathway enrichment and functional annotation were performed using the KEGG database.

### 2.17 Oxygen Consumption Rate Measurement

The OCR was measured using a fluorescence lifetime-based fiber-optic micro-oxygen system (Instech). After treatment for 24 h, cells were resuspended at equal densities. OCR was calculated from the slope of O_2_ concentration over time using linear regression and reported as %O_2_ per minute, normalized to cell number.

### 2.18 *Ex vivo* Xenograft-Derived Explants

Four- to six-week-old female BALB/c nude mice (Charles River Laboratories) were subcutaneously injected with 2×10^6^ HeLa and HepG2 cells suspended in 100 µL of PBS/50% Matrigel. Tumors were harvested at 300-500 mm^3^, rinsed in saline, and cut into 2-3 mm^3^ fragments under sterile conditions. Explants were cultured in 24-well plates with complete DMEM. Tissues were treated for 24 h, then processed for histology and immunohistochemistry 24, 25. For hematoxylin and eosin (H&E) staining, 3 µm sections were stained using a standard kit (Abcam) and imaged under a 20 × objective (Leica DFC310 FX camera). All animal procedures were approved by the Macau Division of Animal Control and Inspection (AL019/DICV/DIS/2025) and conducted in accordance with the guidelines of the Animal Care and Use Committee of Macau University of Science and Technology.

### 2.19 Immunohistochemistry (IHC) Staining

Paraffin-embedded tissue sections were processed for immunohistochemical analysis of cleaved PARP expression. Following deparaffinization, rehydration, and antigen retrieval, sections were incubated overnight with a primary anti-cleaved PARP antibody. Specific binding was detected using a standard biotin-streptavidin-HRP system with DAB chromogen, followed by hematoxylin counterstaining. Representative images were acquired for qualitative assessment of cleaved PARP staining.

### 2.20 Statistical Analysis

All experiments were independently replicated a minimum of three times. Data are reported as mean ± standard deviation (SD) unless specified otherwise. Differences between two groups were assessed using unpaired two-tailed Student's t-tests. For comparisons involving more than two groups, one-way ANOVA was performed, followed by the Student-Newman-Keuls post hoc test. Statistical analyses were conducted using GraphPad Prism 8.0. Significance was defined as *p < 0.05, **p < 0.01, ***p < 0.001, and ****p < 0.0001. All statistical tests applied assumed normal distribution and homogeneity of variance across groups.

## 3. Results

### 3.1 Drug Combination Exerts Synergistic Antiproliferative Effect and Triggers Cellular Energy Crisis

**Figure 1:**
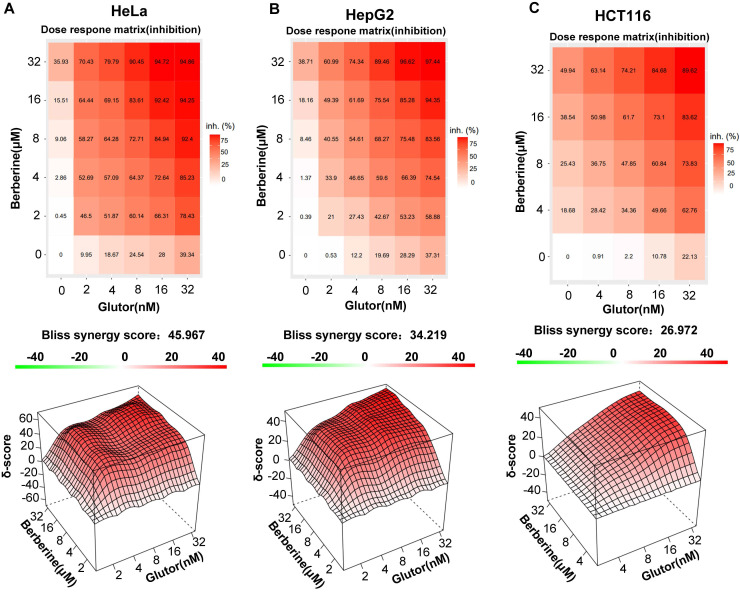
** Synergistic inhibition of tumor cell proliferation by Glutor and BBR.** (A-C) Cell viability was assessed by the SRB assay. Two-dimensional dose-matrix analysis illustrates the anti-proliferative effects of BBR (0-32 µM), Glutor (0-32 nM), alone or in combination, on (A) HeLa, (B) HepG2, and (C) HCT116 cells after 24 or 48 h of treatment. Drug interaction was analyzed using the Bliss independence model with SynergyFinder software. The corresponding 3D synergy plots depict Bliss scores, color-coded to indicate antagonism (green; score < -10), additivity (white; -10 to 10), and synergy (red; score > 10). Data are from three independent experiments; each performed in triplicate. Values are presented as mean ± SD; *p < 0.05, **p < 0.01, ***p < 0.001.

To assess whether BBR in combination with Glutor exerts synergistic antitumor activity and alters cellular energy metabolism, we selected three cancer cell lines with high GLUT1 and GLUT3 expression, i.e., HeLa, HepG2, and HCT116 (Supplementary Fig. 1A). Cell proliferation assays demonstrated that either BBR or Glutor alone suppressed cell growth in a concentration-dependent manner. Synergy analysis using SynergyFinder 3.0 software revealed robust synergy between the two agents, with overall scores of 45.967 in HeLa, 34.219 in HepG2, and 26.972 in HCT116 cells, all substantially exceeding the synergy threshold (score > 10) (Fig. 1A-C). The IC50 values of BBR and Glutor in HeLa, HepG2, and HCT116 cells are provided in Supplementary Table 1. Importantly, across the tested concentration ranges, the combination matrix showed predominantly synergistic antiproliferative effects.

**Figure 2:**
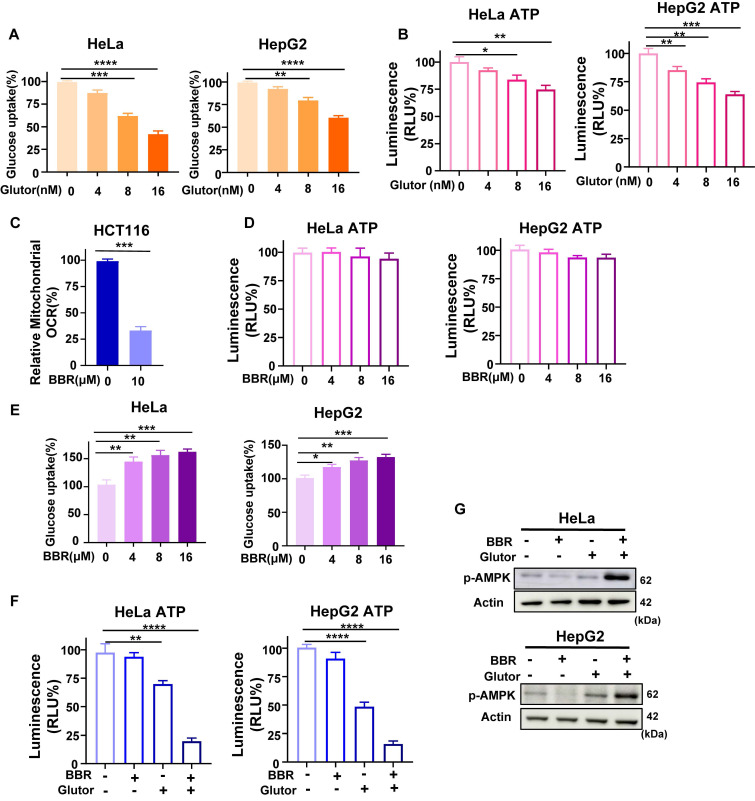
** Glutor and BBR impair bioenergetic metabolism in cancer cells.** (A) Intracellular glucose levels in HeLa and HepG2 cells after 24 h of treatment with Glutor (0-16 nM), as measured using a colorimetric glucose assay. (B) ATP levels in HeLa and HepG2 cells exposed to Glutor (0-16 nM) for 24 h, as determined by the CellTiter-Lumi™ II luminescent assay. (C) Mitochondrial OCR in HCT116 cells measured with a fiber-optic oxygen monitor following BBR treatment. HCT116 was used as a representative model for OCR analysis to validate the inhibitory effect of BBR on mitochondrial respiration. (D) ATP levels in HeLa and HepG2 cells exposed to BBR (0-16 μM) for 24 h, as determined by the CellTiter-Lumi™ II luminescent assay. (E) Intracellular glucose levels in HeLa and HepG2 cells after 24 h of treatment with BBR (0-16 μM), as measured using a colorimetric glucose assay. (F) ATP levels in HeLa and HepG2 cells treated for 24 h with DMSO (vehicle control), BBR (8 μM), Glutor (16 nM), or their combination, as determined by the CellTiter-Lumi™ II luminescent assay. (G) Western blot analysis of p-AMPK protein levels in HeLa and HepG2 cells treated for 24 h with BBR (8 μM) alone, Glutor (16 nM) alone, or their combination. Data are from three independent experiments, each performed in triplicate, and analyzed with GraphPad Prism. Values are presented as mean ± SD; *p < 0.05, **p < 0.01, ***p < 0.001, ****p < 0.0001.

Mechanistically, Glutor suppressed glucose uptake through inhibition of GLUT1/3 7. Consistent with this, Glutor reduced glucose uptake and lactate production in HeLa and HepG2 cells in a dose-dependent manner (Fig. 2A; Supplementary Fig. 1B), and moderately decreased intracellular ATP levels (Fig. 2B). In contrast, BBR alone significantly inhibited mitochondrial respiration in HCT116 cells (Fig. 2C), in agreement with previous reports 26. HCT116 was used here as a representative model for OCR analysis to validate the inhibitory effect of BBR on mitochondrial respiration. Notably, despite suppressing mitochondrial respiration, BBR had little effect on intracellular ATP levels in HeLa and HepG2 cells (Fig. 2D). Meanwhile, BBR alone dose-dependently increased glucose uptake and lactate production in both cell lines (Fig. 2E; Supplementary Fig. 1C), suggesting that under conditions of OXPHOS inhibition, cells may at least partly maintain ATP homeostasis through compensatory enhancement of glycolytic metabolism. To further dissect the combined effects, we selected concentrations of BBR (8 μM) and Glutor (16 nM), which produced only modest single-agent effects on cell proliferation and bioenergetic parameters under the selected conditions. In parallel, we evaluated L02 and AC16 cells under the same working concentrations. Although Glutor alone moderately reduced HeLa and HepG2 cell viability, BBR plus Glutor produced a much stronger reduction, while single-agent or combined treatment caused only slight and non-significant changes in L02 and AC16 cells (Supplementary Fig. 2A, 2B). Under these same working concentrations, the combination treatment significantly reduced intracellular ATP levels relative to the vehicle control and to either monotherapy group (Fig. 2F). This finding underscores that inhibition of glycolysis or OXPHOS alone triggers compensatory metabolic adaptation, whereas simultaneous blockade of both pathways effectively diminishes cellular ATP. Consistent with this interpretation, AMPK phosphorylation was markedly elevated in the combination group, indicating exacerbated bioenergetic stress caused by dual metabolic inhibition (Fig. 2G).

### 3.2 Combination Treatment Triggers Caspase-Dependent Apoptosis

**Figure 3:**
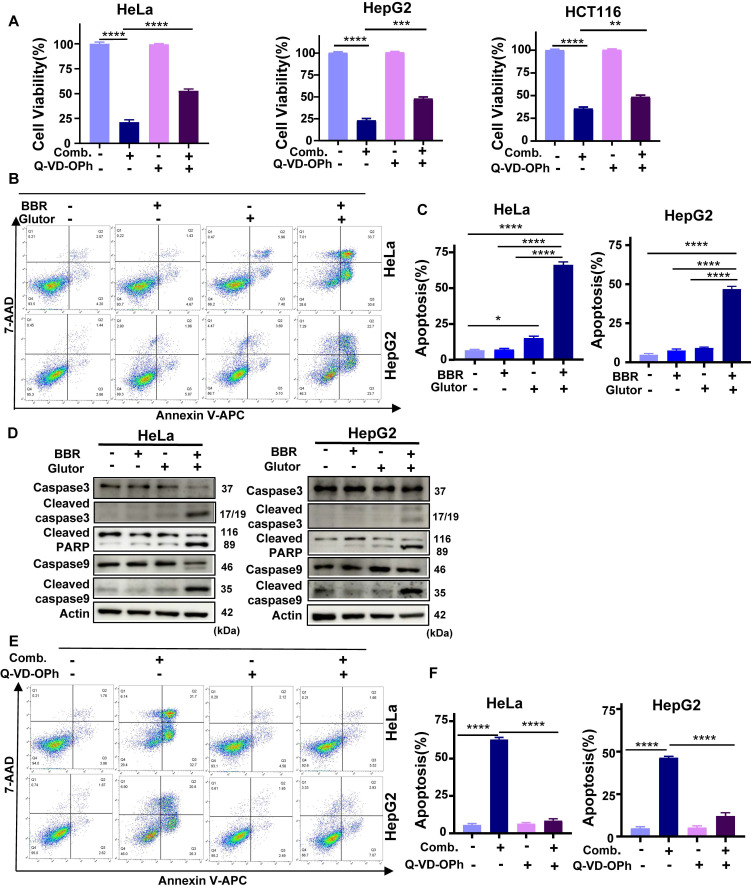
** Combined treatment with BBR and Glutor induces apoptosis, abrogated by Q-VD-OPh.** (A) Cell viability assessed by SRB assay after pretreatment with or without Q-VD-OPh (40 µM), followed by co-treatment with BBR (8 µM) and Glutor (16 nM) for 24 or 48 h. (B, C) Apoptosis in HeLa and HepG2 cells treated for 24 h with DMSO (vehicle control), BBR (8 μM), Glutor (16 nM), or their combination, evaluated by flow cytometry using Annexin V/7-AAD staining. Representative dot plots (B) and quantitative analysis (C) are shown. (D) Western blot analysis of apoptosis-related proteins in HeLa and HepG2 cells treated as in (B). (E, F) Apoptosis in HeLa and HepG2 cells pretreated with or without Q-VD-OPh (40 µM) and then exposed to DMSO (vehicle control) or the BBR-Glutor combination for 24 h, analyzed by flow cytometry (E) and quantified (F). Data are from three independent experiments, each performed in triplicate, and analyzed with GraphPad Prism. Values are presented as mean ± SD; *p < 0.05, **p < 0.01, ***p < 0.001, ****p < 0.0001.

To further delineate the mode of cell death associated with this energy crisis, we pretreated cells with the pan-caspase apoptosis inhibitor Q-VD-OPh, the ferroptosis inhibitors Fer-1 and Lip-1, and the autophagy inhibitor CQ, followed by combined drug administration. The results showed that only Q-VD-OPh markedly reversed the antiproliferative effect of the combination treatment, whereas Fer-1, Lip-1, and CQ exhibited no such rescue activity (Fig. 3A; Supplementary Fig. 2C, 2D). Consistently, colony formation assays demonstrated that Q-VD-OPh pretreatment partially restored the long-term proliferative capacity suppressed by the combination therapy (Supplementary Fig. 3A).

Flow cytometric analysis revealed that the proportion of Annexin V-positive cells was significantly higher in the combination group compared with either monotherapy or control groups (Fig. 3B, 3C). Western blotting further demonstrated that the combined treatment robustly upregulated the levels of key apoptosis execution proteins, including cleaved caspase-3, cleaved caspase-9, and cleaved PARP (Fig. 3D; Supplementary Fig. 3B), in a time-dependent manner (Supplementary Fig. 3C). Given that mitochondrial membrane potential (ΔΨm) depolarization is a critical early event in apoptosis, we assessed ΔΨm using JC-1 staining. The results indicated that the combination treatment significantly induced ΔΨm depolarization in HeLa and HepG2 cells (Supplementary Fig. 3D, 3E). Flow cytometry and Western blot analyses further confirmed that Q-VD-OPh attenuated combination-induced cell death and reduced activation of the aforementioned apoptotic markers (Fig. 3E, 3F; Supplementary Fig. 3F). Taken together, these findings demonstrate that the synergistic antitumor activity of BBR and Glutor is mediated predominantly through the induction of apoptosis.

### 3.3 Enhanced DNA Damage and Impaired Homologous Recombination Repair

**Figure 4:**
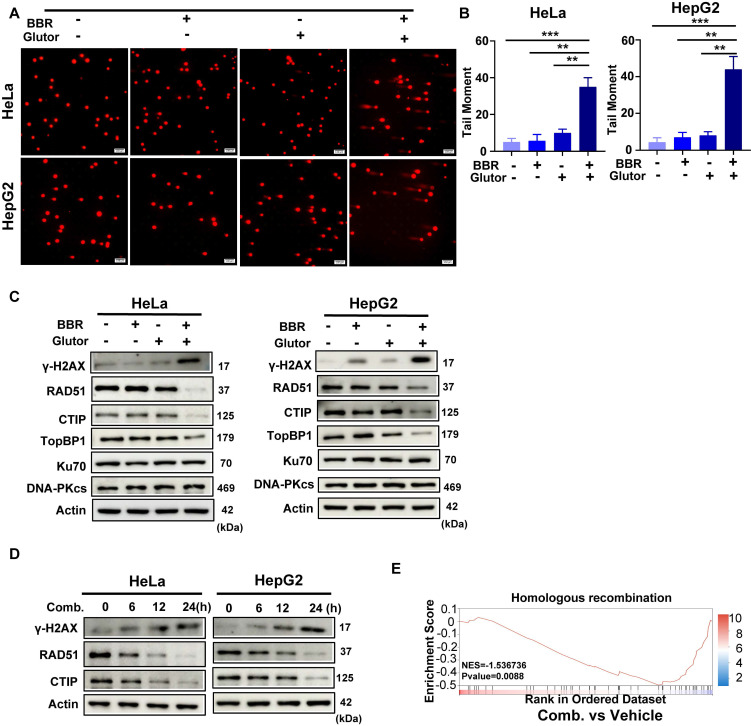
** Combined BBR and Glutor treatment induces DNA damage and HR repair deficiency.** (A, B) Representative images (A) and quantification (B) of neutral comet assays in HeLa and HepG2 cells treated for 24 h with DMSO (vehicle control), BBR (8 μM), Glutor (16 nM), or their combination. (C) Western blot analysis of γ-H2AX, RAD51, CtIP, TopBP1, Ku70, and DNA-PKcs protein levels in HeLa and HepG2 cells treated as described in (A). (D) Time dependent Western blot analysis of γ-H2AX, RAD51, and CtIP protein levels in HeLa and HepG2 cells treated with the BBR- Glutor combination. (E) GSEA revealed a significant downregulation of the homologous recombination pathway in HCT116 cells treated with the combination of BBR (8 μM) and Glutor (16 nM) for 24 h compared to the control group (normalized enrichment score (NES) =-1.537, p = 0.0088). Data are from three independent experiments performed in triplicate and analyzed with GraphPad Prism. Values represent mean ± SD; *p < 0.05, **p < 0.01, ***p < 0.001.

Studies have shown that BBR exerts antitumor effects by inducing DNA damage and promoting apoptosis 27. In the present study, however, under the selected experimental conditions, the most prominent DNA damage phenotype was observed in the combination group. Results from the neutral comet assay demonstrated that the combination treatment induced a significantly higher level of DNA damage compared to either single agent or the control group (Fig. 4A, 4B; Supplementary Fig. 4A, 4B). Consistently, the expression of γ-H2AX, a marker of DNA double-strand breaks, was markedly upregulated upon combined treatment (Fig. 4C, 4D; Supplementary Fig. 4C). To further elucidate how the combination therapy influences DNA damage repair dynamics, we performed proteomic profiling in HCT116 cells. HCT116 cells were used at this discovery stage to define pathway-level changes associated with the combination treatment and to provide a basis for subsequent mechanistic validation in HeLa and HepG2 cells. Gene Set Enrichment Analysis (GSEA) revealed that the combination treatment significantly downregulated gene sets related to homologous recombination repair (Fig. 4E). In agreement with this finding, the expression of key HR repair proteins, including RAD51, CtIP, and TopBP1, was reduced in HeLa and HepG2 cells after combination treatment (Fig. 4C). Time-course analysis further showed progressive γ-H2AX accumulation together with decreased RAD51 and CtIP expression following combination treatment (Fig. 4D). RAD51 protein levels also exhibited a declining trend in HCT116 cells (Supplementary Fig. 4C). In contrast, the expression of key non-homologous end joining (NHEJ) repair proteins, Ku70 and DNA-PKcs, remained largely unaffected (Fig. 4C; Supplementary Fig. 4C). Taken together, the combined treatment of BBR and Glutor enhances its antitumor effect by inducing DNA damage and suppressing HR repair.

### 3.4 JNK Pathway Activation Mediates Synergistic Toxicity

**Figure 5:**
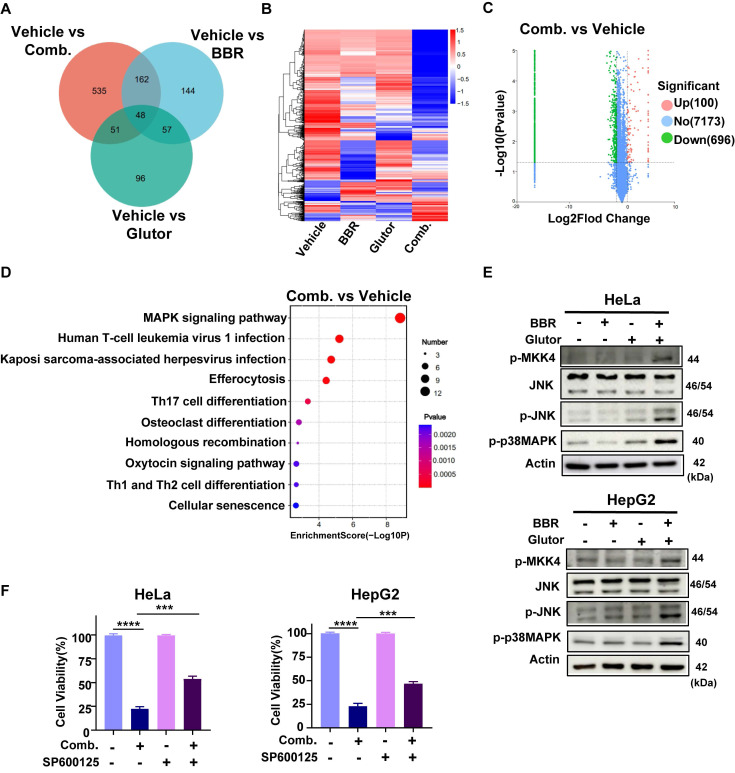
** BBR and Glutor alter the proteomic landscape and activate MAPK signaling.** (A) Venn diagram illustrating the overlap of DEPs (fold change > 2, p < 0.05) in HCT116 cells treated for 24 h with BBR (8 μM), Glutor (16 nM), or their combination, compared to the untreated control. (B) Heatmap of the proteome profile from proteomic analysis, displaying unsupervised clustering. Significant differential expression was defined as |log_2_(FC)| ≥ 1 and p < 0.05. (C) Volcano plot of DEPs between the control and combination treatment groups in HCT116 cells. Upregulated (red) and downregulated (green) proteins are shown (Log10 labeled counts; Fold change (FC) > 2, p < 0.05). (D) KEGG pathway enrichment analysis of DEPs between the combination treatment group and the control. (E) Western blot analysis of p-MKK4, JNK, p-JNK and p-p38 MAPK protein levels in HeLa and HepG2 cells after 24 h treatment with BBR (8 µM), Glutor (16 nM), or their combination. (F) Cell viability (SRB assay) in HeLa and HepG2 cells pretreated with or without SP600125 (4 µM) for 2 h, followed by 24 h treatment with BBR (8 µM) and Glutor (16 nM). Data are from three independent experiments performed in triplicate and analyzed with GraphPad Prism. Values represent mean ± SD; *p < 0.05, **p < 0.01, ***p < 0.001, ****p < 0.0001.

To elucidate the upstream regulatory mechanisms underlying combination-induced DNA damage and apoptosis, proteomic analysis revealed that the combination treatment markedly altered the protein expression profile, with a total of 796 DEPs identified, including 100 upregulated and 696 downregulated proteins (Fig. 5A-C). KEGG pathway enrichment analysis indicated that the MAPK signaling pathway was one of the most significantly affected pathways by the combination treatment (Fig. 5D). To further dissect the involvement of this pathway, we examined the phosphorylation status of key MAPK family proteins in HeLa and HepG2 cells. Western blot analysis demonstrated that the combination treatment robustly increased the phosphorylation of JNK, p38 MAPK and MKK4, with effects substantially stronger than those observed in single-agent or control groups (Fig. 5E). Notably, these phosphorylation events exhibited a time-dependent pattern (Supplementary Fig. 4D). In HCT116 cells, phosphorylation of JNK was likewise markedly elevated (Supplementary Fig. 4E). SRB assays showed that pretreatment with the p38 MAPK inhibitor SB202190 failed to significantly rescue the antiproliferative effect of the combination therapy (Supplementary Fig. 4F), suggesting that p38 MAPK activation might be a concomitant event following cell death initiation. In contrast, pretreatment with the JNK inhibitor SP600125 substantially reduced combination-induced cell death in HeLa and HepG2 cells (Fig. 5F) and attenuated its long-term antiproliferative effects (Supplementary Fig. 5A).

**Figure 6:**
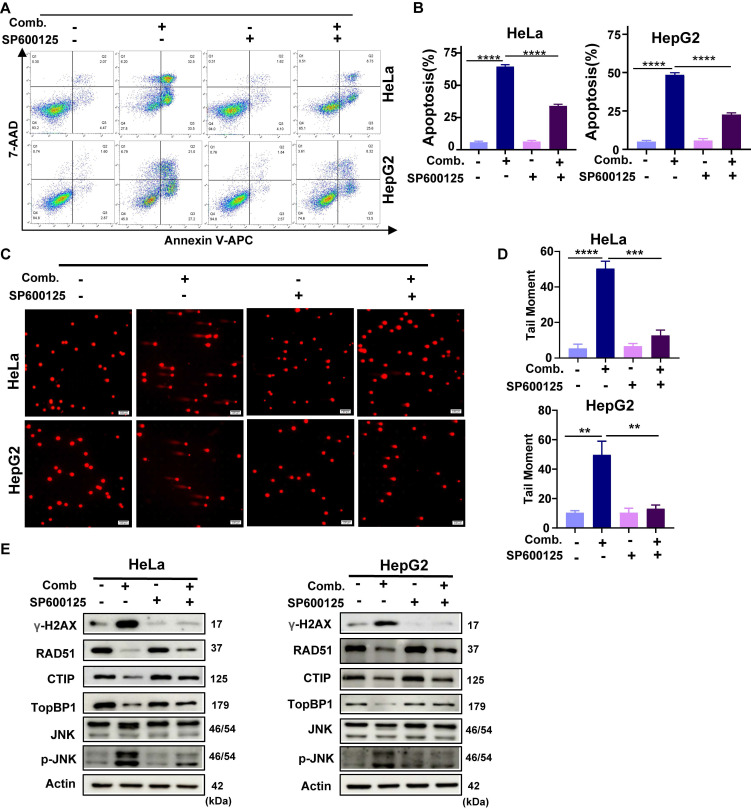
** SP600125 attenuates the antitumor effect of combined BBR and Glutor treatment.** (A, B) Apoptosis assessed by Annexin V/7-AAD flow cytometry after pretreatment with or without the JNK inhibitor SP600125 (4 μM, 2 h), followed by exposure to DMSO (vehicle control) or the BBR-Glutor combination for 24 h. Representative dot plots (A) and quantification (B) are shown. (C, D) Neutral comet assays performed under the same treatment conditions; representative images (C) and quantification of DNA damage (D) are presented. (E) Western blot analysis of γ-H2AX, RAD51, CtIP, TopBP1, JNK and p-JNK, in HeLa and HepG2 cells pretreated with or without SP600125 (4 μM, 2 h) and subsequently exposed to the BBR-Glutor combination for 24 h. Data are from three independent experiments performed in triplicate and analyzed with GraphPad Prism. Values represent mean ± SD; *p < 0.05, **p < 0.01, ***p < 0.001, ****p < 0.0001.

Further analyses revealed that SP600125 mitigated combination-induced apoptosis, ΔΨm depolarization (Fig. 6A, 6B; Supplementary Fig. 5B-D), and DNA damage (Fig. 6C, 6D). Moreover, SP600125 suppressed JNK phosphorylation, reduced the accumulation of the DNA damage marker γ-H2AX, and alleviated the downregulation of HR repair factors (Fig. 6E). As MKK4/7 are direct upstream kinases of JNK, pretreatment with BSJ-04-122, a specific inhibitor of MKK4/7, similarly decreased cellular sensitivity to the combination therapy (Supplementary Fig. 5E). These findings indicate that activation of the JNK signaling pathway is a critical upstream event driving combination-induced DNA damage and cell death.

**Figure 7:**
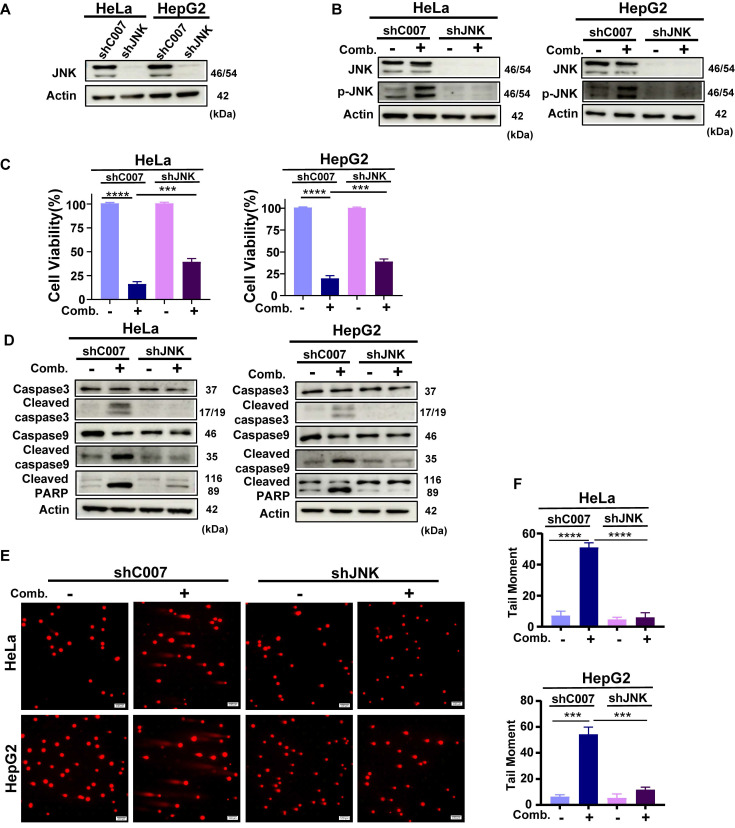
** JNK knockdown reduces the antitumor effect of combined BBR and Glutor treatment.** (A) Western blot analysis of JNK protein expression in HeLa and HepG2 cells transduced with control shRNA (shC007) or JNK-targeting shRNA (shJNK) lentiviruses. (B) Western blot analysis of JNK and p-JNK protein levels in cells transduced with control or JNK-targeting lentiviruses. (C) Cell viability assessed by SRB assay after 24 h treatment with BBR (8 µM) and Glutor (16 nM) in cells transduced with shC007 or shJNK. (D) Western blot analysis of apoptosis-related proteins in shC007- or shJNK-transduced cells treated with the BBR-Glutor combination for 24 h. (E, F) Neutral comet assay performed in control or JNK-silenced HeLa and HepG2 cells after 24 h treatment with DMSO (vehicle control) or the BBR-Glutor combination. Representative fluorescence images (E) and quantification of DNA damage (F) are shown. Data are from three independent experiments performed in triplicate and analyzed with GraphPad Prism. Values represent mean ± SD; *p < 0.05, **p < 0.01, ***p < 0.001, ****p < 0.0001.

To further establish the central role of JNK signaling in the synergistic effect, we generated shRNA-mediated JNK knockdown (JNK-KD) HeLa and HepG2 cell lines. Western blot analysis confirmed that JNK knockdown effectively suppressed the combination-induced phosphorylation of JNK (Fig. 7A, 7B). JNK knockdown significantly rescued the antiproliferative effect of the combination treatment (Fig. 7C). Further analyses revealed that JNK knockdown attenuated combination-induced apoptosis and mitochondrial membrane potential depolarization (Fig. 7D; Supplementary Fig. 6A-D). Additionally, in neutral comet assays, JNK knockdown reduced DNA damage triggered by the combination treatment (Fig. 7E, 7F), concomitant with decreased γ-H2AX protein levels. Moreover, JNK knockdown partially reversed the suppression of key HR repair proteins induced by the combination therapy (Supplementary Fig. 6E). Collectively, these results demonstrate that JNK silencing markedly alleviates combination-induced DNA damage, repair inhibition, and cell death, thereby confirming that activation of the JNK pathway is an indispensable upstream mechanism underlying the synergistic antitumor effect.

### 3.5 *Ex vivo* Validation of Cooperative Antitumor Activity

**Figure 8:**
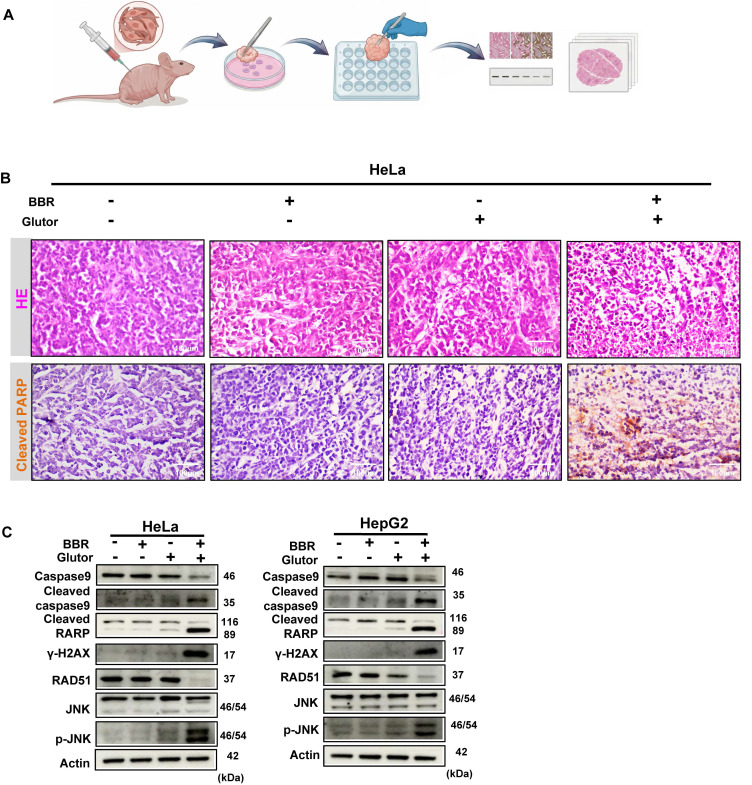
** Combined BBR and Glutor treatment promotes apoptosis and DNA damage in xenograft-derived tumor explants.** (A) Schematic diagram of the experimental workflow for *ex vivo* treatment of HeLa-derived xenograft tumor explants. (B) Histopathology of tumor tissues from HeLa xenografts treated with BBR (8 µM) and/or Glutor (16 nM). Representative images show H&E staining (20×) and IHC staining for cleaved PARP. (C) Western blot analysis of apoptosis-related proteins, DNA damage markers, and JNK/p-JNK in HeLa- and HepG2-derived xenograft tumor explants after 24 h treatment with BBR (8 µM) and/or Glutor (16 nM). Data are representative of three independent experiments.

**Figure 9:**
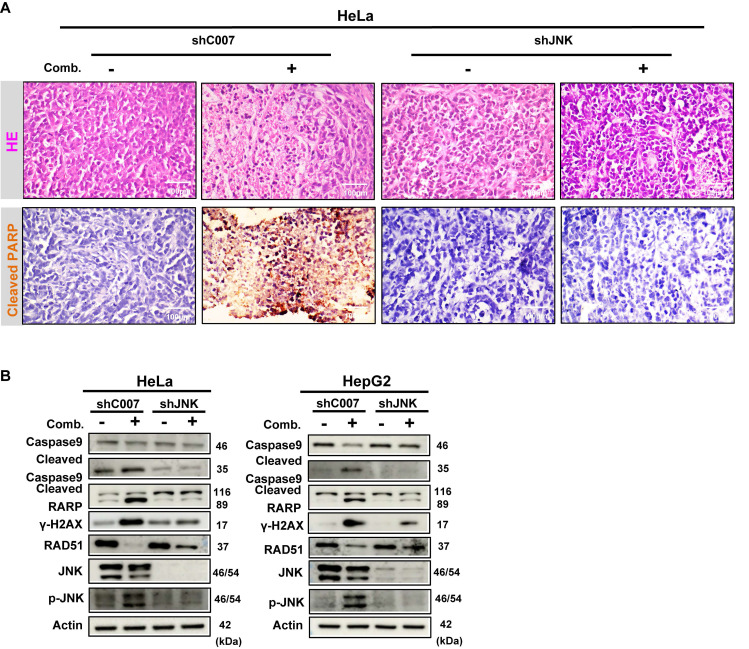
** JNK knockdown attenuates the antitumor effect of combined BBR and Glutor treatment in xenograft-derived tumor explants.** (A) Representative images of H&E staining (20×) and cleaved PARP IHC staining in HeLa-derived xenograft tumor explants transduced with control shRNA (shC007) or JNK-targeting shRNA (shJNK) and treated with the BBR-Glutor combination. (B) Western blot analysis of apoptosis markers, DNA damage markers, and components of the JNK signaling pathway in HeLa- and HepG2-derived xenograft tumor explants transduced with shC007 or shJNK and treated with the BBR-Glutor combination. Data are representative of three independent experiments.

Because Glutor is difficult to obtain in sufficient quantities and no *in vivo* dosing regimen has been reported to date, we employed a xenograft-derived tumor explant culture model as an alternative to conventional *in vivo* xenograft treatment studies to evaluate the efficacy of this combination therapy 28, 29. Since the later mechanistic validation phase was mainly performed in HeLa and HepG2 cells, HeLa-derived explants were used for histological and immunohistochemical tissue-level validation of the major *in vitro* findings. In addition, HepG2-derived explants were used for Western blot-based molecular validation of key signaling and repair markers. HeLa-derived xenograft tumor tissues were sectioned into uniform fragments and subjected to vehicle control, BBR monotherapy, Glutor monotherapy, or combination treatment (Fig. 8A). H&E staining confirmed that the explants retained the morphological characteristics of high-grade carcinoma. Combination treatment induced pronounced histological alterations, with cellular morphology and structural features markedly different from those in the control and monotherapy groups. To assess apoptosis within the xenograft explants, we performed IHC analysis of cleaved PARP. Compared with the control and monotherapy groups, the combination group showed stronger and more widespread cleaved PARP staining, consistent with enhanced apoptotic responses in the explant tissues (Fig. 8B). Consistently, Western blot analysis of HeLa- and HepG2-derived explants showed that combination treatment significantly modulated the expression of key markers associated with JNK signaling, apoptosis, DNA damage, and HR repair (Fig. 8C). To further validate the role of JNK signaling in the combination response at the tissue level, we generated xenograft-derived explants from shC007- or shJNK-transduced HeLa cells and analyzed their responses to BBR-Glutor combination treatment. H&E staining suggested that JNK knockdown reduced the extent of histopathological alterations induced by the combination treatment. IHC results indicated that the combination treatment markedly increased apoptotic cells in the shC007 control group, whereas this pro-apoptotic effect was substantially diminished in the shJNK group (Fig. 9A). Western blot results were consistent with the *in vitro* data (Fig. 9B). In summary, the combination of BBR and Glutor exerts cooperative antitumor effects by activating the JNK signaling pathway, inhibiting HR-mediated DNA repair, promoting DNA damage accumulation, and ultimately inducing tumor cell apoptosis.

## 4. Discussion

In normal cells, energy metabolism generally remains within relatively stable oxidative or glycolytic states. By contrast, cancer cells can adopt a hybrid metabolic phenotype in which glycolysis and OXPHOS are concurrently active, partly as a result of elevated reactive oxygen species (ROS) levels and sustained activation of oncogenic pathways such as RAS, MYC, and c-SRC. Such metabolic plasticity is increasingly recognized as an important contributor to tumor progression and therapeutic resistance. 30. Given the altered reliance of tumor cells on glucose uptake, inhibiting GLUTs has become an important strategy for metabolic intervention. Among such inhibitors, the pan-GLUT inhibitor Glutor significantly reduces glucose uptake in tumor cells by blocking GLUT1/3, leading to decreased glycolytic flux and deficiencies in energy and biosynthetic precursors. When combined with the glutaminase inhibitor CB-839, Glutor disrupts the glucose-glutamine metabolic axis, resulting in NADPH depletion, ROS accumulation, and collapse of the MYC/GLS1 regulatory circuit, thereby effectively suppressing glycolysis-dependent tumors 3, 31. Consistent with these reports, our experiments confirmed that Glutor monotherapy significantly reduces glucose uptake and lactate production in HeLa and HepG2 cells, indicating suppressed glycolysis. However, tumor cells possess strong metabolic adaptability and may compensate for glucose limitation by enhancing the oxidative metabolism of alternative substrates such as glutamine and fatty acids.

On the other hand, BBR exhibits broad metabolic regulatory and antitumor activities. Our previous studies revealed that BBR can interfere with OXPHOS by inhibiting mitochondrial complex I. Particularly in IDH1-mutated acute myeloid leukemia, the combination of BBR with the IDH1 inhibitor AG-120 synergistically enhanced anti-leukemic efficacy 32. In our current study, BBR significantly reduced the OCR of cells, while ATP levels did not change markedly, suggesting that tumor cells may compensate for OXPHOS inhibition by enhancing glycolysis. Importantly, the combination of BBR and Glutor led to a sharp decline in ATP levels, with effects significantly superior to either agent alone. This combined treatment simultaneously inhibits glucose uptake and mitochondrial OXPHOS, thereby dually blocking the energy supply of tumor cells, effectively overcoming their metabolic compensatory capacity, and inducing a bioenergetic crisis and cell death.

The present findings also distinguish the BBR-Glutor combination from the previously reported BBR + 2-DG strategy. In the earlier study, the synergistic effect was primarily linked to ATP depletion and UPR disruption, and AMPK activation was found to be dispensable 19. By contrast, our data identify persistent AMPK activation, JNK-dependent DNA damage signaling, suppression of HR repair, and apoptosis as key downstream events associated with the BBR-Glutor combination. This difference is mechanistically meaningful because 2-DG acts after glucose uptake and is known to affect processes beyond glycolysis, including N-linked glycosylation and ER stress 33, 34, whereas Glutor blocks glucose entry at the transporter level. In addition, the clinical and translational development of 2-DG as an anticancer agent has been constrained by limited single-agent efficacy, the need for relatively high exposure, and safety liabilities observed in both clinical and preclinical studies 35-37.

AMPK is a central regulator of cellular energy metabolism and can respond to energy stress through dual classical and non-classical mechanisms. The classical pathway is activated by sensing changes in the AMP/ATP ratio, while the non-classical lysosomal pathway independently senses glucose availability via the FBP-aldolase axis. These two pathways enable AMPK to initiate metabolic reprogramming and amplify the response during an energy crisis 38. In this study, combined treatment with BBR and Glutor significantly enhanced AMPK phosphorylation, with effects superior to either single agent, suggesting that the combination is most consistent with predominant activation of the classical AMPK pathway in response to energy stress. We propose that the two drugs likely act synergistically to reduce intracellular ATP levels, thereby strongly activating AMPK signaling, which in turn reprograms cellular metabolism, inhibits anabolism, and promotes catabolism, ultimately impairing tumor cell viability. This view is supported by previous studies. For instance, in gemcitabine-treated pancreatic cancer cells, although mitochondrial OXPHOS was suppressed, ATP was still preferentially used to activate the AMP-cAMP axis, thereby inhibiting tumor growth via AMPK and PKA pathways 39. *Inonotus obliquus* polysaccharides, by activating the LKB1/AMPK axis and concurrently inhibiting both OXPHOS and glycolysis, induced ATP depletion and triggered mitochondrial-dependent apoptosis, thereby exerting anti-lung cancer effects 40. Our study shows that the antitumor effects of BBR and Glutor in HeLa and HepG2 cells are closely associated with AMPK activation, suggesting that AMPK may serve as an important mediator of the combination treatment. Taken together, our data are most consistent with a working model in which profound bioenergetic stress and subsequent AMPK/JNK activation precede the more pronounced accumulation of DNA damage and HR-repair suppression, although partial temporal overlap among these events cannot be excluded.

Based on proteomic analysis, we found that the MAPK signaling pathway was significantly enriched following combined treatment with BBR and Glutor, suggesting its important role in the drug combination-induced cell death process. Among its members, JNK, a key component of the MAPK family 41, plays a context-dependent dual role in cancer, acting either as an "oncogenic engine" or a "tumor-suppressive guardian" 42. In various cancers, JNK can function to stabilize and activate tumor properties, promoting progression and enhancing chemoresistance 43-46; however, its inactivation can also facilitate tumorigenesis 47. Under specific stress conditions, its activation can instead induce apoptosis 48-51. Notably, JNK activation is often coupled with AMPK signaling. For example, in liver cancer, pseudolaric acid B activates AMPK via energy stress, subsequently upregulates JNK, and induces mitochondrial fission and apoptosis through p-DRP1 52; extracellular polysaccharide EPS1-1 from *Rhizopus nigricans* activates AMPK via ROS and LKB1, cooperatively inhibits mTORC1 and activates the JNK-p53 axis, driving apoptosis in colon cancer cells 53; in prostate cancer, AICAR induces apoptosis through the AMPK-ROS-JNK-caspase-3 cascade 54. Conversely, fig leaf extract can alleviate pancreatic β-cell apoptosis by inhibiting the AMPK/JNK pathway 55. These studies suggest that under energy stress conditions, AMPK and JNK can form a co-regulated tumor-suppressive signaling axis. Consistent with these mechanisms, this study found that combined treatment with BBR and Glutor significantly activated the JNK signaling pathway. More importantly, pharmacological intervention using the JNK inhibitor SP600125 markedly attenuated the antiproliferative and cell-death effects of the combination treatment, functionally confirming that JNK activation is a necessary mechanism for this combined strategy. In summary, our findings suggest that the AMPK/JNK signaling axis may represent an important mechanistic link underlying the synergistic antitumor effects of BBR and Glutor, providing additional insight into metabolic stress-mediated antitumor strategies.

Activation of the JNK signaling pathway not only regulates energy stress and apoptosis but is also closely involved in the DNA damage response and repair processes 56. Studies indicate that JNK can influence DNA damage in multiple ways. For instance, in hepatocellular carcinoma, inhibiting the JNK/ATM/ATR axis can confer tolerance to DNA damage 57; in non-small cell lung cancer, metabolic stress induced by deficiency in the polyol pathway can activate JNK signaling, upregulate transcription factors such as c-Jun and ATF3, thereby exacerbating DNA damage and driving irreversible cell death 58. Notably, JNK itself can also directly phosphorylate histone H2AX, thus participating directly in the propagation of DNA damage signals and the regulation of apoptosis 59, 60. This mechanism has been studied in various cancer models: in bladder urothelial carcinoma, ALKBH8 inhibits tumor growth and invasion by activating the JNK/p38/γ-H2AX pathway in a NOX-1-dependent ROS manner 61; in chronic myeloid leukemia K562 cells, resveratrol promotes H2AX phosphorylation and induces apoptosis by activating the p38/JNK pathway 62. Our study similarly found that combined treatment with BBR and Glutor significantly activated JNK signaling, induced DNA damage, and elevated γ-H2AX protein levels, consistent with the aforementioned reports. DNA double-strand breaks are primarily repaired through two pathways: HR and non-homologous end joining 63. The MAPK signaling pathway plays an important regulatory role in HR repair 64. Research has shown that deep-sea water can inhibit skin carcinogenesis by activating the AMPK/mTOR and JNK/c-Jun pathways and downregulating the expression of the key HR protein RAD51 65. In the present study, we further demonstrated that the combination of BBR and Glutor can activate the JNK signaling pathway, induce DNA damage, and inhibit HR repair, ultimately leading to tumor cell death. These results support the JNK-DNA damage/HR repair axis as a key downstream mechanism contributing to the synergistic antitumor effect of this combination regimen. Taken together, these findings support a model in which BBR and Glutor functionally cooperate by restricting two partially compensatory bioenergetic pathways 66-68. Glutor suppressed glucose uptake and glycolytic activity, whereas BBR impaired mitochondrial bioenergetics and, in the representative OCR model, decreased mitochondrial respiration without markedly lowering ATP, consistent with compensatory adaptation under single-agent treatment. In contrast, the combination caused profound ATP depletion together with enhanced AMPK phosphorylation, JNK-associated DNA damage signaling, and apoptosis, supporting the view that dual pathway restriction overwhelms compensatory metabolic adaptation under the tested conditions. However, because substrate-limited extracellular flux analysis, isotope tracing, and metabolomic profiling were not performed, we cannot formally determine whether this interaction represents non-additive metabolic coupling at the network level 69.

This study revealed synergistic antiproliferative effects of BBR and Glutor in multiple tumor cell lines, yet several limitations remain. Glutor remains a preclinical GLUT inhibitor, and its *in vivo* dosing regimen, administration route, pharmacokinetic exposure, and systemic safety profile have not yet been established, which restricts the translational interpretation of the present findings. Importantly, although the present study included a preliminary *in vitro* evaluation in L02 and AC16 cells, these models cannot fully represent highly energy-dependent normal tissues or rapidly proliferating normal cell populations. Thus, the therapeutic window, systemic tolerability, and potential on-target toxicities of this strategy remain to be determined in future *in vivo* studies. Comprehensive animal toxicity assessment, including body-weight monitoring, hematological and serum biochemical analyses, and major-organ histopathology, was not performed in the present study and should be included in future *in vivo* studies. In addition, although our data link the synergistic effect of BBR and Glutor to bioenergetic stress, enhanced AMPK phosphorylation, JNK activation, DNA-damage accumulation, and HR-repair suppression, the precise temporal relationship between ATP depletion/AMPK activation and DNA damage has not been rigorously resolved. We therefore cannot exclude partial overlap among these events, nor can we rule out a direct contribution of severe ATP depletion to impaired DNA repair. Moreover, the cell lines employed in this study predominantly exhibit high GLUT expression and may not fully represent the metabolic heterogeneity of diverse tumor types. Finally, the lack of flux-based and metabolomic analyses limits our ability to determine whether the combination induces non-additive metabolic coupling beyond enhanced ATP depletion. Future studies should extend validation to additional tumor types and explore toxicity-mitigation approaches, such as tumor-targeted delivery, stimuli-responsive nanocarrier systems, prodrug strategies, or intermittent dosing to enhance tumor selectivity and tolerability 70-74.

## 5. Conclusion

This study demonstrates that the combination of BBR and Glutor exerts a pronounced synergistic antitumor effect. Mechanistically, the two agents cooperatively suppress glycolysis and OXPHOS, thereby imposing a dual blockade on tumor cell metabolism. This metabolic inhibition activates the AMPK/JNK signaling cascade, which in turn exacerbates DNA damage and impairs HR repair, ultimately leading to effective induction of apoptosis. Collectively, these findings provide experimental evidence supporting further preclinical evaluation and translational development of BBR and Glutor co-administration as a dual metabolic inhibition strategy.

## Supplementary Material

Supplementary figures.

## Data Availability

Data will be made available on request.

## References

[1] Faubert B, Solmonson A, DeBerardinis RJ (2020). Metabolic reprogramming and cancer progression. Science.

[2] Yadav D, Yadav A, Bhattacharya S, Dagar A, Kumar V, Rani R (2024). GLUT and HK: Two primary and essential key players in tumor glycolysis. Semin Cancer Biol.

[3] Reckzeh ES, Karageorgis G, Schwalfenberg M, Ceballos J, Nowacki J, Stroet MCM (2019). Inhibition of Glucose Transporters and Glutaminase Synergistically Impairs Tumor Cell Growth. Cell Chem Biol.

[4] Temre MK, Yadav S, Goel Y, Pandey SK, Kumar A, Singh SM (2022). Glutor, a Glucose Transporter Inhibitor, Exerts Antineoplastic Action on Tumor Cells of Thymic Origin: Implication of Modulated Metabolism, Survival, Oxidative Stress, Mitochondrial Membrane Potential, pH Homeostasis, and Chemosensitivity. Front Oncol.

[5] Momcilovic M, Bailey ST, Lee JT, Fishbein MC, Braas D, Go J (2018). The GSK3 Signaling Axis Regulates Adaptive Glutamine Metabolism in Lung Squamous Cell Carcinoma. Cancer Cell.

[6] Guo L, Zhang W, Xie Y, Chen X, Olmstead EE, Lian M (2022). Diaminobutoxy-substituted Isoflavonoid (DBI-1) Enhances the Therapeutic Efficacy of GLUT1 Inhibitor BAY-876 by Modulating Metabolic Pathways in Colon Cancer Cells. Mol Cancer Ther.

[7] Katt WP, Lukey MJ, Cerione RA (2019). Starving the Devourer: Cutting Cancer Off from Its Favorite Foods. Cell Chem Biol.

[8] Zhou W, Asif A, Situ C, Wang J, Hao H (2025). Multiple target and regulatory pathways of berberine. Phytomedicine.

[9] Habtemariam S (2020). Berberine pharmacology and the gut microbiota: A hidden therapeutic link. Pharmacol Res.

[10] Chen G, Zhang C, Zou J, Zhou Z, Zhang J, Yan Y (2025). Coptidis rhizoma and berberine as anti-cancer drugs: A 10-year updates and future perspectives. Pharmacol Res.

[11] Lin X, Zhang J, Chu Y, Nie Q, Zhang J (2024). Berberine prevents NAFLD and HCC by modulating metabolic disorders. Pharmacol Ther.

[12] Sun Y, Yu J, Liu X, Zhang C, Cao J, Li G (2018). Oncosis-like cell death is induced by berberine through ERK1/2-mediated impairment of mitochondrial aerobic respiration in gliomas. Biomed Pharmacother.

[13] Kwon S, Chan AT (2020). Extracting the benefits of berberine for colorectal cancer. Lancet Gastroenterol Hepatol.

[14] Mori S, Fujiwara-Tani R, Gyoten M, Nukaga S, Sasaki R, Ikemoto A (2023). Berberine Induces Combined Cell Death in Gastrointestinal Cell Lines. Int J Mol Sci.

[15] Abrams SL, Akula SM, Meher AK, Steelman LS, Gizak A, Duda P (2021). GSK-3β Can Regulate the Sensitivity of MIA-PaCa-2 Pancreatic and MCF-7 Breast Cancer Cells to Chemotherapeutic Drugs, Targeted Therapeutics and Nutraceuticals. Cells.

[16] Li W, Li D, Kuang H, Feng X, Ai W, Wang Y (2020). Berberine increases glucose uptake and intracellular ROS levels by promoting Sirtuin 3 ubiquitination. Biomed Pharmacother.

[17] Sajeev A, Sailo B, Unnikrishnan J, Talukdar A, Alqahtani MS, Abbas M (2024). Unlocking the potential of Berberine: Advancing cancer therapy through chemosensitization and combination treatments. Cancer Lett.

[18] Ponnusamy L, Kothandan G, Manoharan R (2020). Berberine and Emodin abrogates breast cancer growth and facilitates apoptosis through inactivation of SIK3-induced mTOR and Akt signaling pathway. Biochim Biophys Acta Mol Basis Dis.

[19] Fan LX, Liu CM, Gao AH, Zhou YB, Li J (2013). Berberine combined with 2-deoxy-d-glucose synergistically enhances cancer cell proliferation inhibition via energy depletion and unfolded protein response disruption. Biochim Biophys Acta.

[20] Wang Y, Sun Z, Zhao Z, Pang J, Chen J (2025). Recent Progress in the Development of Glucose Transporter (GLUT) Inhibitors. J Med Chem.

[21] McGuirk S, Audet-Delage Y, St-Pierre J (2020). Metabolic Fitness and Plasticity in Cancer Progression. Trends Cancer.

[22] Huang XM, Huang JJ, Du JJ, Zhang N, Long Z, Yang Y (2021). Autophagy inhibitors increase the susceptibility of KRAS-mutant human colorectal cancer cells to a combined treatment of 2-deoxy-D-glucose and lovastatin. Acta Pharmacol Sin.

[23] Ianevski A, Giri AK, Aittokallio T (2022). SynergyFinder 3.0: an interactive analysis and consensus interpretation of multi-drug synergies across multiple samples. Nucleic Acids Res.

[24] Wu Q, Ba-Alawi W, Deblois G, Cruickshank J, Duan S, Lima-Fernandes E (2020). GLUT1 inhibition blocks growth of RB1-positive triple negative breast cancer. Nat Commun.

[25] Adams CM, Mitra R, Xiao Y, Michener P, Palazzo J, Chao A (2023). Targeted MDM2 Degradation Reveals a New Vulnerability for p53-Inactivated Triple-Negative Breast Cancer. Cancer Discov.

[26] Yu M, Alimujiang M, Hu L, Liu F, Bao Y, Yin J (2021). Berberine alleviates lipid metabolism disorders via inhibition of mitochondrial complex I in gut and liver. Int J Biol Sci.

[27] Hou D, Xu G, Zhang C, Li B, Qin J, Hao X (2017). Berberine induces oxidative DNA damage and impairs homologous recombination repair in ovarian cancer cells to confer increased sensitivity to PARP inhibition. Cell Death Dis.

[28] Venkata PP, Chen Y, Alejo S, He Y, Palacios BE, Loeffel I (2022). KDM1A inhibition augments the efficacy of rapamycin for the treatment of endometrial cancer. Cancer Lett.

[29] Nagle AM, Levine KM, Tasdemir N, Scott JA, Burlbaugh K, Kehm J (2018). Loss of E-cadherin Enhances IGF1-IGF1R Pathway Activation and Sensitizes Breast Cancers to Anti-IGF1R/InsR Inhibitors. Clin Cancer Res.

[30] Yu L, Lu M, Jia D, Ma J, Ben-Jacob E, Levine H (2017). Modeling the Genetic Regulation of Cancer Metabolism: Interplay between Glycolysis and Oxidative Phosphorylation. Cancer Res.

[31] Temre MK, Kumar A, Singh SM (2022). An appraisal of the current status of inhibition of glucose transporters as an emerging antineoplastic approach: Promising potential of new pan-GLUT inhibitors. Front Pharmacol.

[32] Huang Z, Shen Y, Liu W, Yang Y, Guo L, Yan Q (2023). Berberine targets the electron transport chain complex I and reveals the landscape of OXPHOS dependency in acute myeloid leukemia with IDH1 mutation. Chin J Nat Med.

[33] Kurtoglu M, Gao N, Shang J, Maher JC, Lehrman MA, Wangpaichitr M (2007). Under normoxia, 2-deoxy-D-glucose elicits cell death in select tumor types not by inhibition of glycolysis but by interfering with N-linked glycosylation. Mol Cancer Ther.

[34] Xi H, Kurtoglu M, Liu H, Wangpaichitr M, You M, Liu X (2011). 2-Deoxy-D-glucose activates autophagy via endoplasmic reticulum stress rather than ATP depletion. Cancer Chemother Pharmacol.

[35] Xi H, Kurtoglu M, Lampidis TJ (2014). The wonders of 2-deoxy-D-glucose. IUBMB Life.

[36] Raez LE, Papadopoulos K, Ricart AD, Chiorean EG, Dipaola RS, Stein MN (2013). A phase I dose-escalation trial of 2-deoxy-D-glucose alone or combined with docetaxel in patients with advanced solid tumors. Cancer Chemother Pharmacol.

[37] Fokt I, Cybulski M, Skora S, Pająk B, Ziemniak M, Woźniak K (2023). d-Glucose- and d-mannose-based antimetabolites. Part 4: Facile synthesis of mono- and di-acetates of 2-deoxy-d-glucose prodrugs as potentially useful antimetabolites. Carbohydr Res.

[38] Lin SC, Hardie DG (2018). AMPK: Sensing Glucose as well as Cellular Energy Status. Cell Metab.

[39] Liu J, Jing W, Wang T, Hu Z, Lu H (2023). Functional metabolomics revealed the dual-activation of cAMP-AMP axis is a novel therapeutic target of pancreatic cancer. Pharmacol Res.

[40] Jiang S, Shi F, Lin H, Ying Y, Luo L, Huang D (2020). Inonotus obliquus polysaccharides induces apoptosis of lung cancer cells and alters energy metabolism via the LKB1/AMPK axis. Int J Biol Macromol.

[41] Wagner EF, Nebreda AR (2009). Signal integration by JNK and p38 MAPK pathways in cancer development. Nat Rev Cancer.

[42] Wu Q, Wu W, Fu B, Shi L, Wang X, Kuca K (2019). JNK signaling in cancer cell survival. Med Res Rev.

[43] Chen X, Hao A, Li X, Ye K, Zhao C, Yang H (2020). Activation of JNK and p38 MAPK Mediated by ZDHHC17 Drives Glioblastoma Multiforme Development and Malignant Progression. Theranostics.

[44] Cui C, Zhang H, Yang C, Yin M, Teng X, Yang M (2024). Inhibition of JNK Signaling Overcomes Cancer-Associated Fibroblast-Mediated Immunosuppression and Enhances the Efficacy of Immunotherapy in Bladder Cancer. Cancer Res.

[45] Zhou W, Xu Y, Zhang J, Zhang P, Yao Z, Yan Z (2022). MiRNA-363-3p/DUSP10/JNK axis mediates chemoresistance by enhancing DNA damage repair in diffuse large B-cell lymphoma. Leukemia.

[46] Vasilevskaya IA, Selvakumaran M, Hierro LC, Goldstein SR, Winkler JD, O'Dwyer PJ (2015). Inhibition of JNK Sensitizes Hypoxic Colon Cancer Cells to DNA-Damaging Agents. Clin Cancer Res.

[47] Itah Z, Chaudhry S, Raju Ponny S, Aydemir O, Lee A, Cavanagh-Kyros J (2023). HER2-driven breast cancer suppression by the JNK signaling pathway. Proc Natl Acad Sci U S A.

[48] Shi S, Tian X, Gong Y, Sun M, Liu J, Zhang J (2024). Pivotal role of JNK protein in the therapeutic efficacy of parthenolide against breast cancer: Novel and comprehensive evidences from network pharmacology, single-cell RNA sequencing and metabolomics. Int J Biol Macromol.

[49] Olszewski K, Barsotti A, Feng XJ, Momcilovic M, Liu KG, Kim JI (2022). Inhibition of glucose transport synergizes with chemical or genetic disruption of mitochondrial metabolism and suppresses TCA cycle-deficient tumors. Cell Chem Biol.

[50] Jin H, Liu C, Liu X, Wang H, Zhang Y, Liu Y (2024). Huaier suppresses cisplatin resistance in non-small cell lung cancer by inhibiting the JNK/JUN/IL-8 signaling pathway. J Ethnopharmacol.

[51] Liu J, Wang T, Creighton CJ, Wu SP, Ray M, Janardhan KS (2019). JNK(1/2) represses Lkb(1)-deficiency-induced lung squamous cell carcinoma progression. Nat Commun.

[52] Liu Z, Wang N, Meng Z, Lu S, Peng G (2023). Pseudolaric acid B triggers cell apoptosis by activating AMPK/JNK/DRP1/mitochondrial fission pathway in hepatocellular carcinoma. Toxicology.

[53] Lu Y, Zhang X, Wang J, Chen K (2020). Exopolysaccharides isolated from Rhizopus nigricans induced colon cancer cell apoptosis in vitro and in vivo via activating the AMPK pathway. Biosci Rep.

[54] Sauer H, Engel S, Milosevic N, Sharifpanah F, Wartenberg M (2012). Activation of AMP-kinase by AICAR induces apoptosis of DU-145 prostate cancer cells through generation of reactive oxygen species and activation of c-Jun N-terminal kinase. Int J Oncol.

[55] Zhang Y, Li Y, Ma P, Chen J, Xie W (2020). Ficus carica leaves extract inhibited pancreatic β-cell apoptosis by inhibiting AMPK/JNK/caspase-3 signaling pathway and antioxidation. Biomed Pharmacother.

[56] Roos WP, Kaina B (2006). DNA damage-induced cell death by apoptosis. Trends Mol Med.

[57] Liu C, Lin X, Sun B, Mao Z, Chen L, Qian H (2021). PRCC reduces the sensitivity of cancer cells to DNA damage by inhibiting JNK and ATM/ATR pathways and results in a poor prognosis in hepatocellular carcinoma. Cell Biosci.

[58] Schwab A, Siddiqui MA, Ramesh V, Gollavilli PN, Turtos AM, Møller SS (2025). Polyol pathway-generated fructose is indispensable for growth and survival of non-small cell lung cancer. Cell Death Differ.

[59] Lu C, Zhu F, Cho YY, Tang F, Zykova T, Ma WY (2006). Cell apoptosis: requirement of H2AX in DNA ladder formation, but not for the activation of caspase-3. Mol Cell.

[60] Sluss HK, Davis RJ (2006). H2AX is a target of the JNK signaling pathway that is required for apoptotic DNA fragmentation. Mol Cell.

[61] Shimada K, Nakamura M, Anai S, De Velasco M, Tanaka M, Tsujikawa K (2009). A novel human AlkB homologue, ALKBH8, contributes to human bladder cancer progression. Cancer Res.

[62] Wu XP, Xiong M, Xu CS, Duan LN, Dong YQ, Luo Y (2015). Resveratrol induces apoptosis of human chronic myelogenous leukemia cells in vitro through p38 and JNK-regulated H2AX phosphorylation. Acta Pharmacol Sin.

[63] Chowdhury D, Choi YE, Brault ME (2013). Charity begins at home: non-coding RNA functions in DNA repair. Nat Rev Mol Cell Biol.

[64] Golding SE, Rosenberg E, Neill S, Dent P, Povirk LF, Valerie K (2007). Extracellular signal-related kinase positively regulates ataxia telangiectasia mutated, homologous recombination repair, and the DNA damage response. Cancer Res.

[65] Lee KS, Lee MG, Woo YJ, Nam KS (2019). The preventive effect of deep sea water on the development of cancerous skin cells through the induction of autophagic cell death in UVB-damaged HaCaT keratinocyte. Biomed Pharmacother.

[66] Hay N (2016). Reprogramming glucose metabolism in cancer: can it be exploited for cancer therapy?. Nat Rev Cancer.

[67] Gui DY, Sullivan LB, Luengo A, Hosios AM, Bush LN, Gitego N (2016). Environment Dictates Dependence on Mitochondrial Complex I for NAD+ and Aspartate Production and Determines Cancer Cell Sensitivity to Metformin. Cell Metab.

[68] Birsoy K, Wang T, Chen WW, Freinkman E, Abu-Remaileh M, Sabatini DM (2015). An Essential Role of the Mitochondrial Electron Transport Chain in Cell Proliferation Is to Enable Aspartate Synthesis. Cell.

[69] Vasan K, Werner M, Chandel NS (2020). Mitochondrial Metabolism as a Target for Cancer Therapy. Cell Metab.

[70] Rosenblum D, Joshi N, Tao W, Karp JM, Peer D (2018). Progress and challenges towards targeted delivery of cancer therapeutics. Nat Commun.

[71] Sun L, Liu H, Ye Y, Lei Y, Islam R, Tan S (2023). Smart nanoparticles for cancer therapy. Signal Transduct Target Ther.

[72] Shen H, Zhang C, Li S, Liang Y, Lee LT, Aggarwal N (2024). Prodrug-conjugated tumor-seeking commensals for targeted cancer therapy. Nat Commun.

[73] Fourie Zirkelbach J, Shah M, Vallejo J, Cheng J, Ayyoub A, Liu J (2022). Improving Dose-Optimization Processes Used in Oncology Drug Development to Minimize Toxicity and Maximize Benefit to Patients. J Clin Oncol.

[74] Shah M, Rahman A, Theoret MR, Pazdur R (2021). The Drug-Dosing Conundrum in Oncology - When Less Is More. N Engl J Med.

