# Supplementary material for: Dual Metabolic Inhibition by Berberine and Glutor Triggers AMPK/JNK-Dependent DNA Damage in Cancer Cells
Source: Int J Med Sci. 2026 May 29;23(7):2355–75. doi: 10.7150/ijms.132977 (PMC13280739; doi:10.7150/ijms.132977)
Supplement: Supplementary file 1 — Supplementary figures. [file ijmsv23p2355s1.pdf]

Supplementary Information

Supplementary Table 1 IC50 values of BBR and Glutor in different cell lines.

Cell line	Treatment duration	BBR IC50 (μM)	Glutor IC50 (nM)
HeLa	24 h	43.72	38.24
HepG2	24 h	51.73	49.91
HCT116	48 h	31.27	106.80

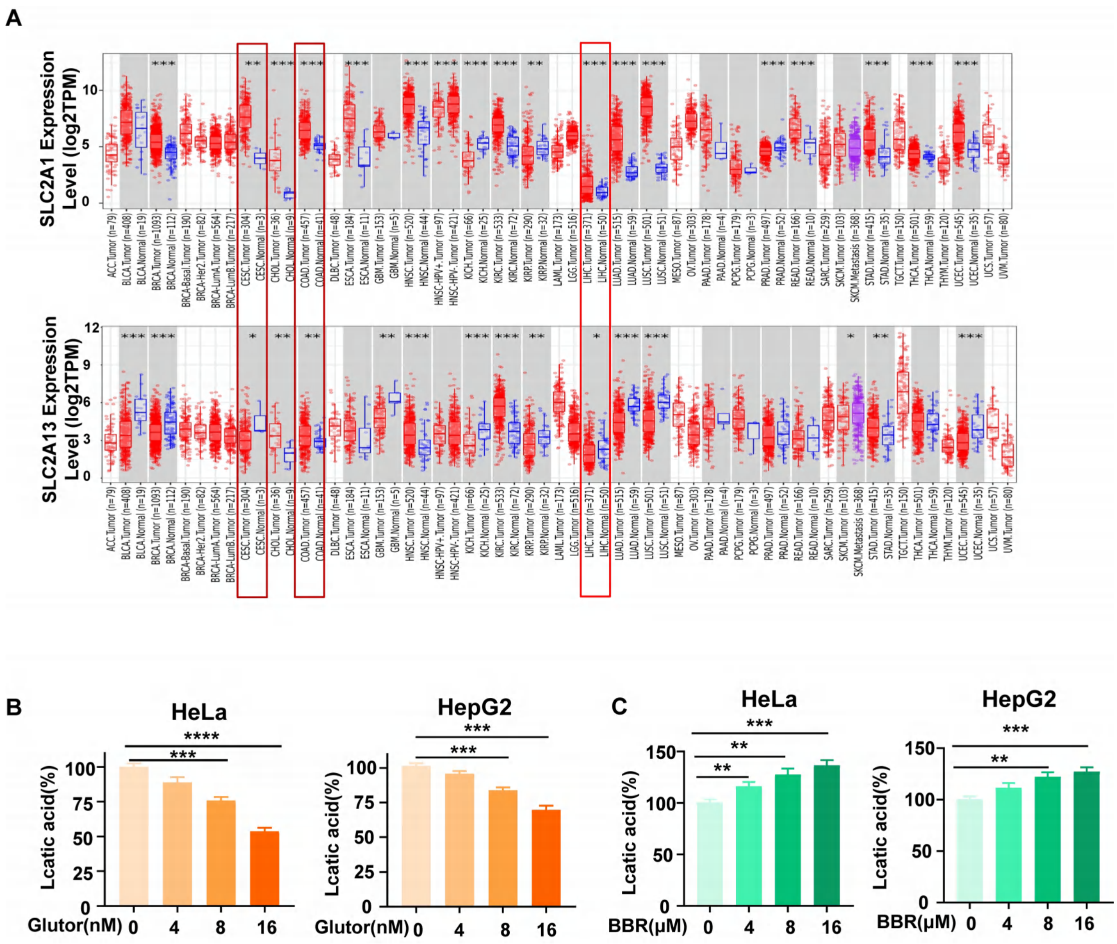

Supplementary Figure 1. GLUT1/GLUT3 expression and lactate changes related to BBR or Glutor treatment.

(A) Analysis of SLC2A1 (GLUT1) and SLC2A3 (GLUT3) expression in tumor versus matched adjacent normal tissues across Cervical and Endocervical Cancer (CESC), Liver Hepatocellular Carcinoma (LIHC), and colon adenocarcinoma (COAD) from The Cancer Genome Atlas (TCGA). The cancer types relevant to this study are highlighted with red boxes. (B) Intracellular lactate levels measured by L-lactate colorimetric assay in HeLa and HepG2 cells after 24 h treatment with Glutor (0–16 nM). (C) Lactate production in HeLa and HepG2 cells after 24 h treatment with BBR (0-16 μM). Data are presented as mean ± SD from three independent experiments performed in triplicate. Statistical analysis was performed using GraphPad Prism; *p < 0.05, **p < 0.01, ***p < 0.001, ****p < 0.0001.

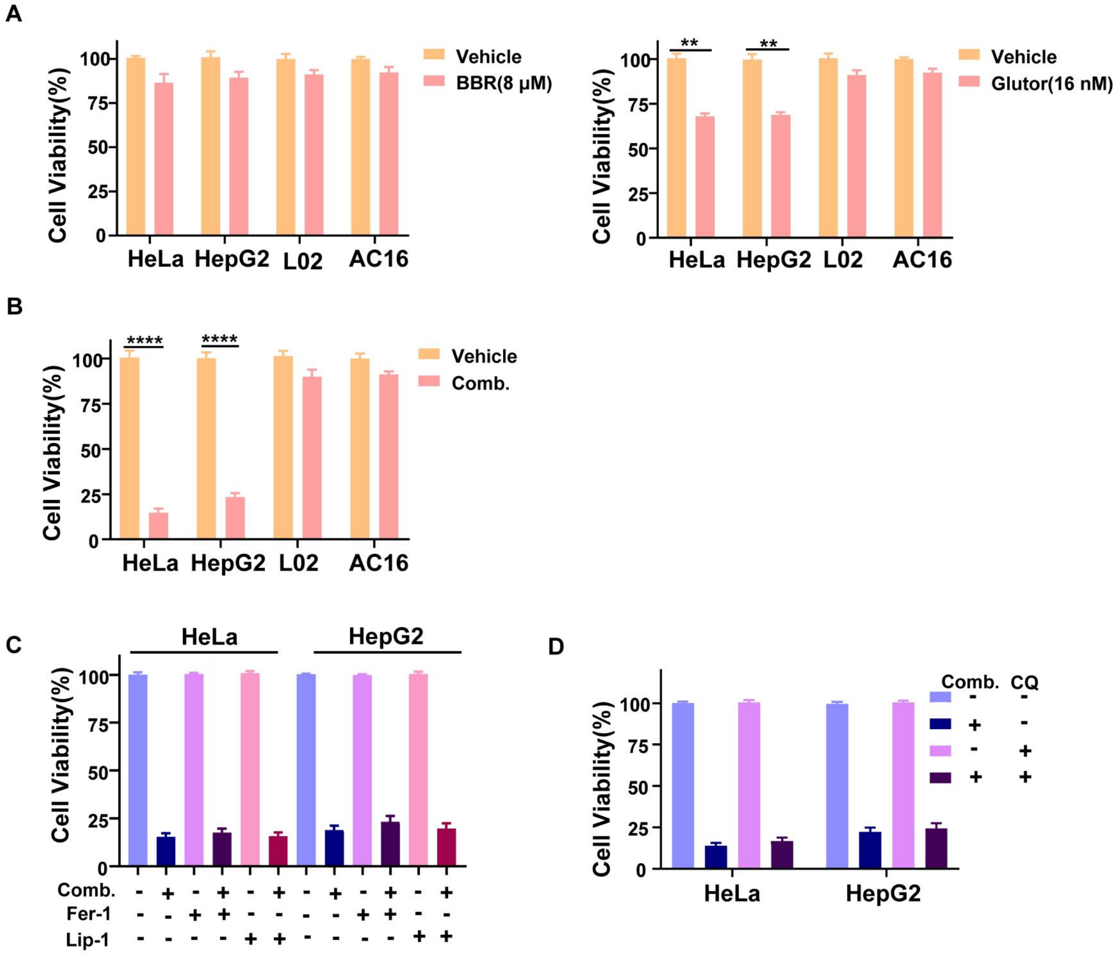

Supplementary Figure 2. Cell viability was assessed under the indicated treatment conditions. (A, B) HeLa, HepG2, L02, and AC16 cells were treated with BBR (8 μM), Glutor (16 nM), or their combination for 24 h, and cell viability was determined using the SRB assay. Vehicle-treated cells were used as controls. (C, D) Cell viability (SRB assay) of HeLa and HepG2 cells pretreated with or without (C) Fer-1 (2 μM) or Lip-1 (2 μM), or (D) CQ (10 μM), followed by 24 h co-treatment with BBR (8 μM) and Glutor (16 nM). Data are presented as mean ± SD from three independent experiments performed in triplicate. Statistical analysis was performed using GraphPad Prism; *$p < 0.05$, **$p < 0.01$, ***$p < 0.001$, ****$p < 0.0001$.

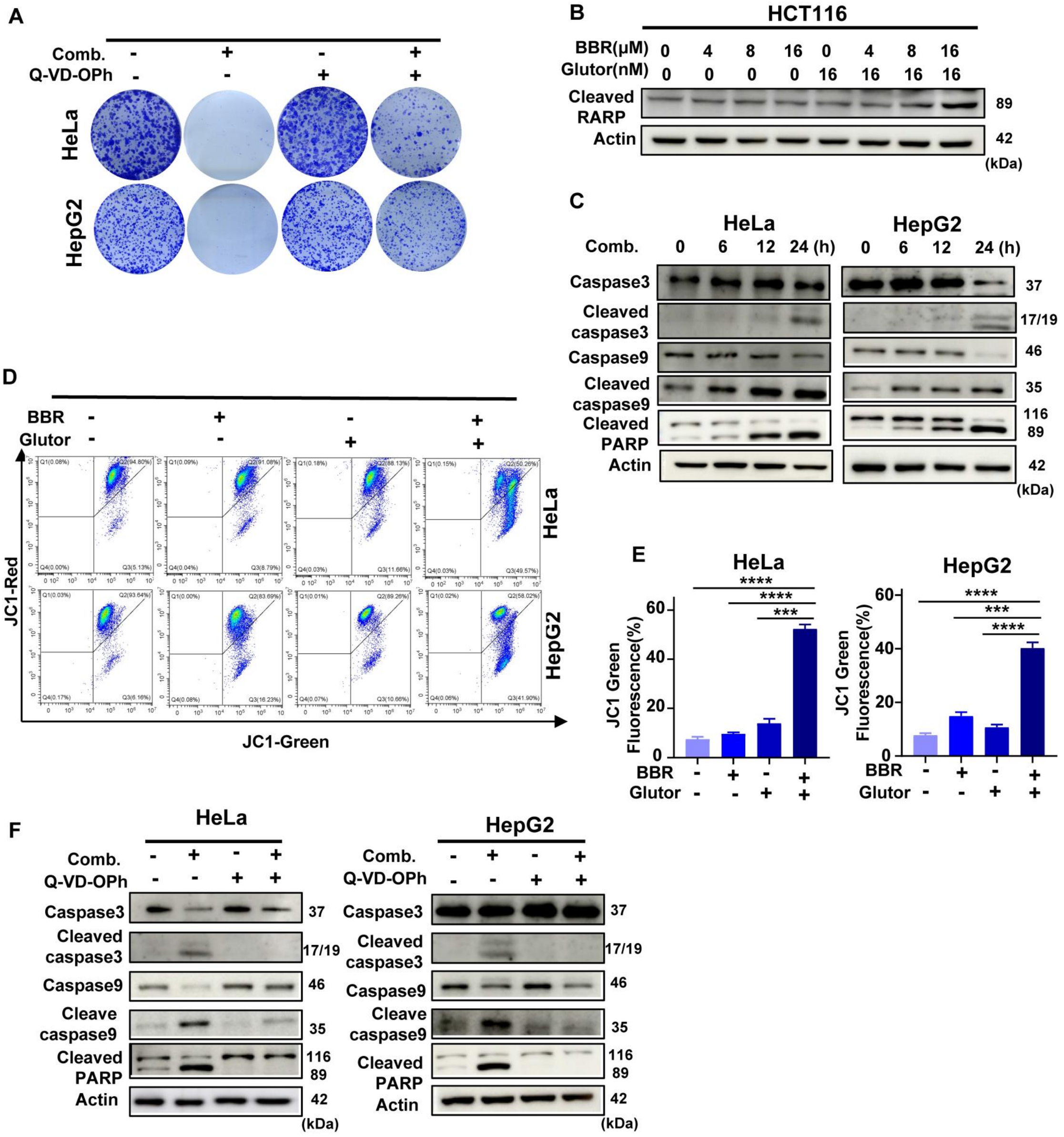

Supplementary Figure 3. Antitumor Effects of Combined BBR and Glutor Treatment.

(A) HeLa and HepG2 cells were cultured for 2 days after seeding, pretreated with Q-VD-OPh, and then treated with BBR (4 μM) plus Glutor (4 nM) for 8 days, followed by colony formation analysis. (B) Western blot analysis of cleaved PARP protein levels in HCT116 cells after 48 h treatment with increasing concentrations of BBR (0–16 μM), Glutor (16 nM), or their combination. (C) Time-course Western blot analysis of apoptosis-related proteins in HeLa and HepG2 cells treated with the BBR (8 μM) and Glutor (16 nM) combination. (D, E) ΔΨm assessed by JC-1 staining and flow cytometry in HeLa and HepG2 cells after 24 h treatment with BBR (8 μM), Glutor (16 nM), or their combination. Representative dot plots (D) and quantification (E) are shown. (F) Western blot analysis of apoptosis-related proteins in HeLa and HepG2 cells pretreated with or without Q-VD-OPh (40 μM)

before 24 h co-treatment with BBR (8 μM) and Glutor (16 nM). Data are presented as mean ± SD from three independent experiments performed in triplicate. Statistical analysis was performed using GraphPad Prism; *p < 0.05, **p < 0.01, ***p < 0.001, ****p < 0.0001.

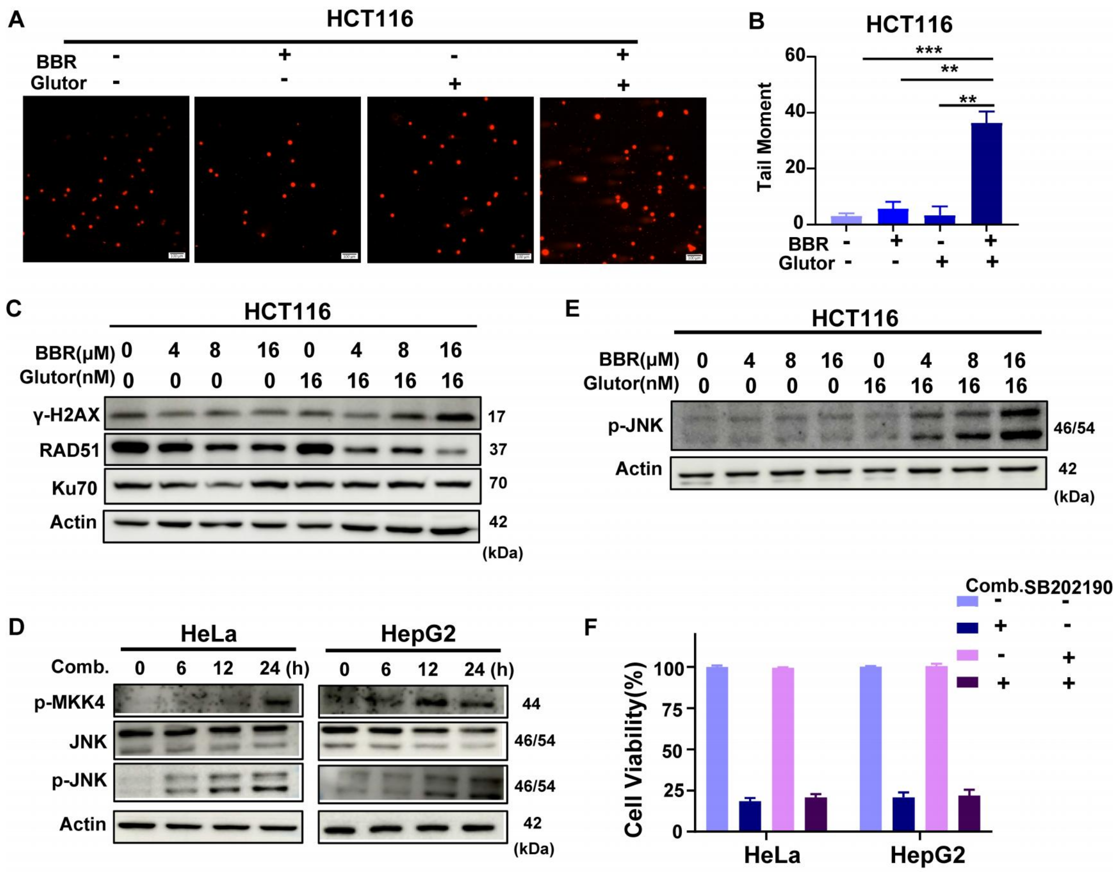

Supplementary Figure 4. BBR combined with Glutor induces DNA damage and activates JNK signaling.

(A, B) DNA damage assessed by neutral comet assay in HCT116 cells after 48 h treatment with DMSO (vehicle control), BBR (8 μM), Glutor (16 nM), or their combination. Representative images (A) and quantification (B) are shown. (C) Western blot analysis of γ-H2AX, RAD51, and Ku70 protein levels in HCT116 cells after 48 h treatment with increasing concentrations of BBR, Glutor (16 nM), or their combination. (D) Time-dependent Western blot analysis of p-MKK4, JNK and p-JNK in HeLa and HepG2 cells after combination treatment with BBR (8 μM) and Glutor (16 nM). (E) Western blot analysis of p-JNK in HCT116 cells after 48 h treatment with increasing concentrations of BBR, Glutor (16 nM), or their combination. (F) Cell viability (SRB assay) in HeLa and HepG2 cells pretreated for 2 h with or without the p38MAPK inhibitor SB202190 (2.5 μM), followed by 24 h combination treatment with BBR (8 μM) and Glutor (16 nM). Data are presented as mean ± SD from three independent experiments performed in triplicate. Statistical analysis was performed using GraphPad Prism; *p < 0.05, **p < 0.01, ***p < 0.001.

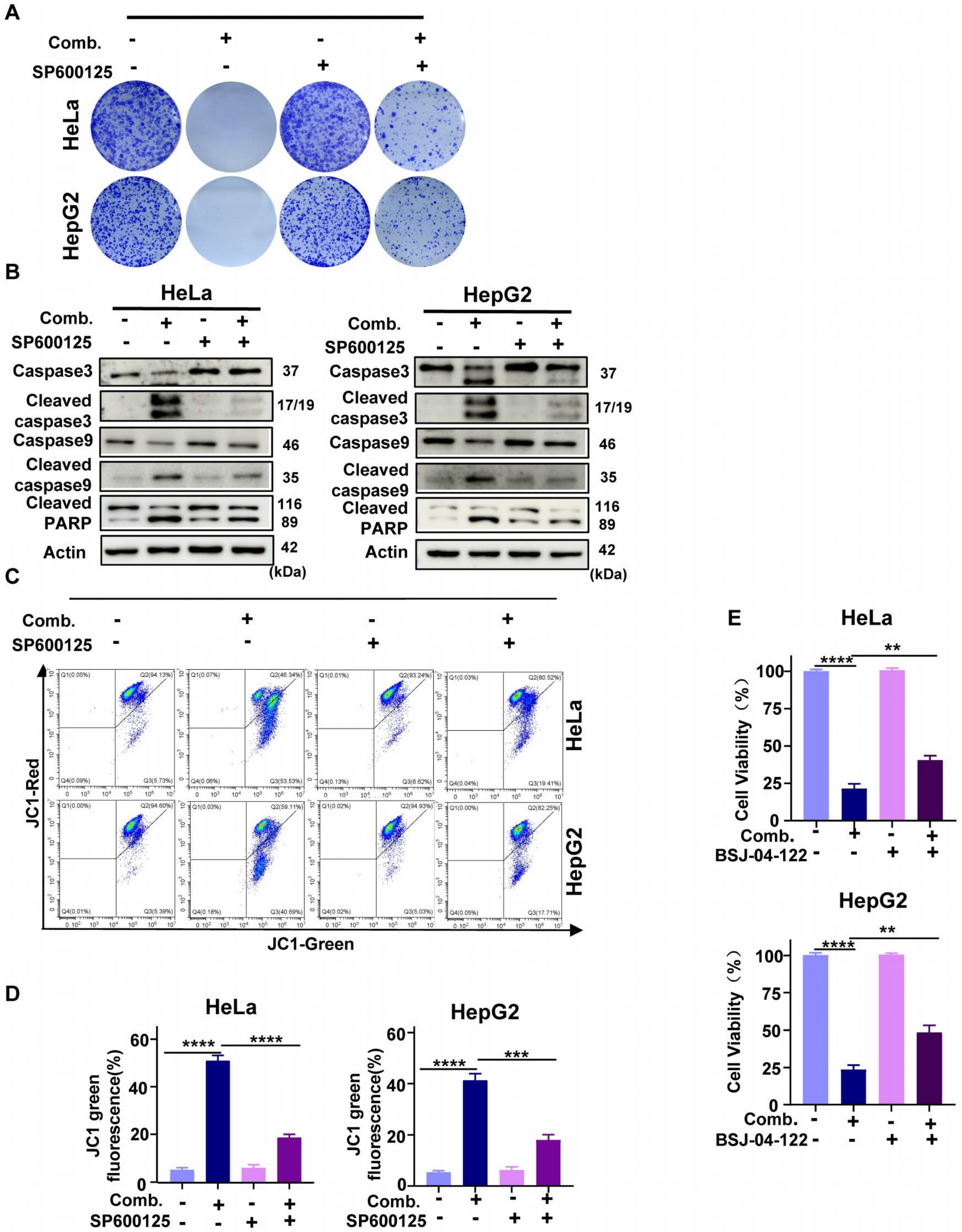

Supplementary Figure 5. Pharmacological inhibition of JNK signaling attenuates BBR–Glutor-induced cell death.

(A) Colony formation assay of HeLa and HepG2 cells pretreated with SP600125 (4 µM) and then treated for 8 days with BBR (4 µM) and Glutor (4 nM). (B) Western blot analysis of apoptosis-related proteins in HeLa and HepG2 cells pretreated with SP600125 before combination treatment with BBR and Glutor. (C, D) Mitochondrial membrane potential assessed by JC-1 staining and flow cytometry in HeLa and HepG2 cells pretreated with or without SP600125 (4 µM, 2 h) followed by 24 h combination treatment. Representative dot plots (C) and quantification (D) are shown. (E) Cell viability (SRB assay)

in HeLa and HepG2 cells pretreated with or without BSJ-04-122 (2 μM) before 24 h cotreatment with BBR (8 μM) and Glutor (16 nM). Data are presented as mean ± SD from three independent experiments performed in triplicate. Statistical analysis was performed using GraphPad Prism; $^*p < 0.05$, $^{**}p < 0.01$, $^{***}p < 0.001$, $^{****}p < 0.0001$.

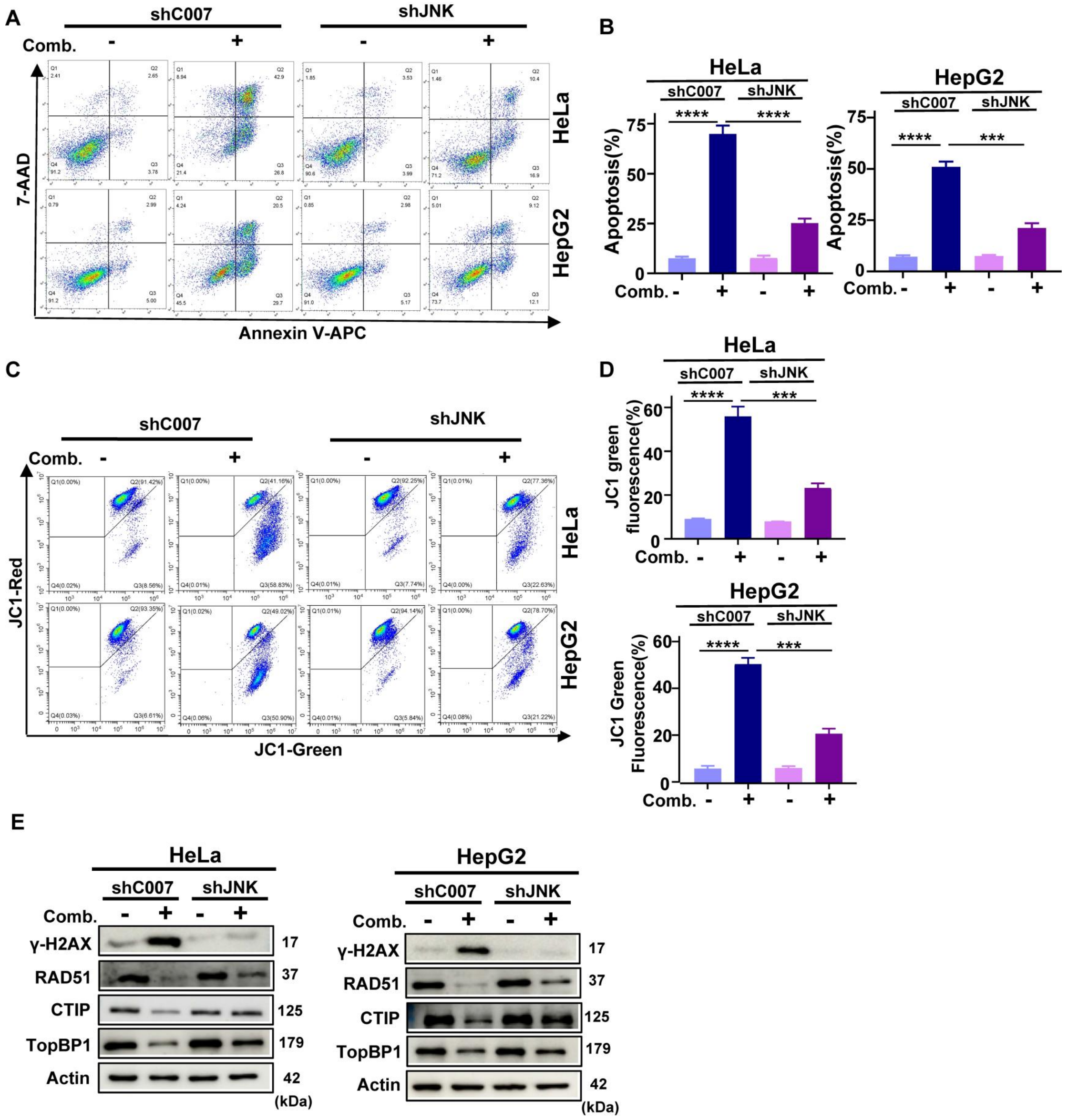

Supplementary Figure 6. JNK knockdown attenuates BBR–Glutor-induced cytotoxic responses. (A, B) Apoptosis assessed by Annexin V/7-AAD staining and flow cytometry in HeLa and HepG2 cells transduced with control (shC007) or JNK-targeting (shJNK) shRNA and treated for 24 h with DMSO (vehicle control) or the BBR–Glutor combination. Representative dot plots (A) and quantification (B) are shown. (C, D) ΔΨm assessed by JC-1 staining and flow cytometry in control or JNK-silenced HeLa and HepG2 cells after 24 h combination treatment with BBR (8 μM) and Glutor (16 nM). Representative dot plots (C) and quantification (D) are displayed. (E) Western blot analysis of γ-H2AX, RAD51, CtIP, and TopBP1 protein levels in HeLa and HepG2 cells transduced with shC007 or shJNK and treated with the BBR–Glutor combination for 24 h. Data are presented as mean ± SD from three independent experiments performed in triplicate. Statistical analysis was performed using GraphPad Prism; *p < 0.05, **p < 0.01, ***p < 0.001, ****p < 0.0001.
